# Effect of Dimensionality on Photoluminescence and Dielectric Properties of Imidazolium Lead Bromides

**DOI:** 10.1021/acs.inorgchem.2c02496

**Published:** 2022-09-14

**Authors:** Szymon Smółka, Mirosław Mączka, Dawid Drozdowski, Dagmara Stefańska, Anna Gągor, Adam Sieradzki, Jan K. Zaręba, Maciej Ptak

**Affiliations:** † Institute of Low Temperature and Structure Research, Polish Academy of Sciences, ul. Okólna 2, 50-422 Wrocław, Poland; ‡ Department of Experimental Physics, Wrocław University of Science and Technology, Wybrzeże Wyspiańskiego 27, 50-370 Wrocław, Poland; § Institute of Advanced Materials, Faculty of Chemistry, Wrocław University of Science and Technology, Wybrzeże Wyspiańskiego 27, 50-370 Wrocław, Poland

## Abstract

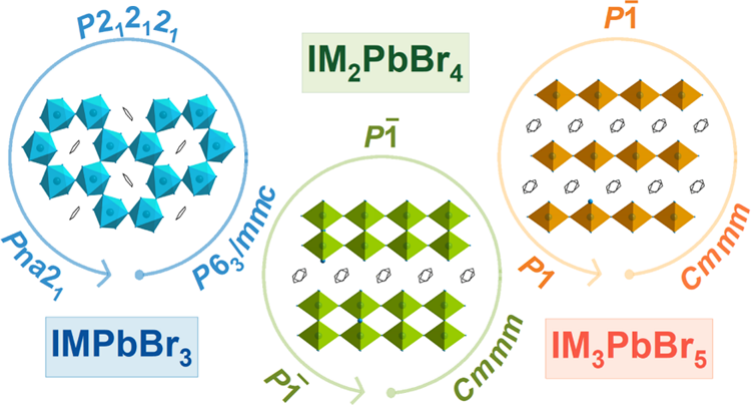

Hybrid organic–inorganic lead halide perovskites have emerged as promising materials for various applications, including solar cells, light-emitting devices, dielectrics, and optical switches. In this work, we report the synthesis, crystal structures, and linear and nonlinear optical as well as dielectric properties of three imidazolium lead bromides, IMPbBr_3_, IM_2_PbBr_4_, and IM_3_PbBr_5_ (IM^+^ = imidazolium). We show that these compounds exhibit three distinct structure types. IMPbBr_3_ crystallizes in the 4H-hexagonal perovskite structure with face- and corner-shared PbBr_6_ octahedra (space group *P*6_3_/*mmc* at 295 K), IM_2_PbBr_4_ adopts a one-dimensional (1D) double-chain structure with edge-shared octahedra (space group *P*1̅ at 295 K), while IM_3_PbBr_5_ crystallizes in the 1D single-chain structure with corner-shared PbBr_6_ octahedra (space group *P*1̅ at 295 K). All compounds exhibit two structural phase transitions, and the lowest temperature phases of IMPbBr_3_ and IM_3_PbBr_5_ are noncentrosymmetric (space groups *Pna*2_1_ at 190 K and *P*1 at 100 K, respectively), as confirmed by measurements of second-harmonic generation (SHG) activity. X-ray diffraction and thermal and Raman studies demonstrate that the phase transitions feature an order–disorder mechanism. The only exception is the isostructural *P*1̅ to *P*1̅ phase transition at 141 K in IM_2_PbBr_4_, which is of a displacive type. Dielectric studies reveal that IMPbBr_3_ is a switchable dielectric material, whereas IM_3_PbBr_5_ is an improper ferroelectric. All compounds exhibit broadband, highly shifted Stokes emissions. Features of these emissions, *i.e.*, band gap and excitonic absorption, are discussed in relation to the different structures of each composition.

## Abstract 2

Three imidazolium lead bromides of various chemical compositions and crystal structures display broadband photoluminescence that can be tuned from bluish-green to orange. All compounds exhibit two structural phase transitions, which lead to interesting optical and electrical properties such SHG activity, ferroelectricity, or dielectric switching.

## Introduction

In recent years, the field of hybrid organic–inorganic perovskites has become one of the most promising research directions for a variety of optoelectronic applications like solar cells,^1−3^ lasers,^4^ and light-emitting devices.^5,6^ The general chemical formula of three-dimensional (3D) hybrid halide perovskites is ABX_3_, in which A is an organic cation, B denotes an inorganic cation (*e.g.*, Pb^2+^, Sn^2+^), and X is a halide anion (Cl^–^, Br^–^, I^–^). Three-dimensional (3D) lead halide perovskites were reported for only four organic cations: methylammonium (MA^+^),^4,7,8^ formamidinium (FA^+^),^4,7−9^ methylhydrazinium (MHy^+^),^10−12^ and aziridinium.^13^ The most well-known MAPbI_3_ and FAPbI_3_ exhibit convenient solution processability as well as great optical and electrical properties for solar cell applications like narrow band gaps, high carrier mobility, and strong light absorption.^6,9^ On the other hand, recently discovered 3D MHyPbBr_3_ exhibits four kinds of functional properties such as photoluminescence (PL), second-harmonic generation (SHG), two-photon excited PL, and switchable dielectric property, whereas MHyPbCl_3_ shows PL and quadratic nonlinear optical (NLO) switching between two SHG-on states.^10,11^

Since the 3D lead halide perovskites can be constructed for only a few organic cations, a lot of attention has been paid to the low-dimensional analogues, which also exhibit unique optoelectronic parameters, but surpass their 3D counterparts in terms of resistance to moisture and irradiation stresses.^14−16^

There is no denying that for photovoltaic applications iodide-based perovskites are more prospective than bromide analogues mostly because of their narrow band gaps. Nevertheless, bromide-based perovskites, both 3D and two-dimensional (2D), were found to be attractive NLO^11,17−19^ and ferroelectric^18,20^ materials. Furthermore, the desirability of lead bromide perovskites also comes from their interesting emission properties. For light-emitting devices, diverse distribution of emitting wavelengths and intense PL is required. The extant literature describes plenty of ways of creating wavelength-tunable materials.^16^ One possibility of tuning the emission wavelength is to synthesize lead halide perovskites comprising two different halides, like in MAPbI_3–*x*_Br*_x_* or MHyPbBr_3–*x*_Cl*_x_*.^21,22^ Another way is to decrease the dimensionality to 2D, one-dimensional (1D), or zero-dimensional (0D)^23,24^ or manipulate the thickness of the inorganic slabs in 2D perovskites by changing the number of inorganic bromide layers (*n*), like in (RNH_3_)_2_(MA)_*n*−1_Pb*_n_*Br_3*n*+1_ (RNH_3_ = long organic ammonium cation).^25^ It is worth mentioning that the type and energy of emissions both depend on the crystal structure. In this respect, three different types of PL are recognized in lead halide perovskites, *i.e*., broadband PL with a large Stokes shift assigned to self-trapped excitons (STEs), relatively narrow PL with a small Stokes shift due to bound excitons (BEs), and a narrow PL explained by free excitons (FEs).^16,19,26−28^ Narrow PL attributed to FE and BE states is typically observed for 3D perovskites and 2D analogues with the crystallographic orientation ⟨100⟩,^29,30^ while broadband STE-related PL often occurs in corrugated 2D structures (⟨110⟩ and ⟨111⟩),^14,30^ as well as in 1D and 0D structures.^23,31−33^ However, PL related to STE states can also be observed for some ⟨100⟩-oriented perovskites. For instance, both FE- and STE-type PL was reported for (PMA)_2_PbBr_4_ (PMA = phenylmethylammonium) and MHy_2_PbBr_4_.^18,34^

It is worth mentioning that due to the hydrophobic properties of imidazolium derivatives, they are used for improving the hydrophobic properties of lead halide perovskites and thus their resistance to moisture.^35−37^ Small IM^+^ cations can also be doped into a 3D perovskite structure, enhancing the efficiency of solar cells.^38^ Regarding imidazolium lead bromides, only one compound was reported in the literature with the chemical composition IMPbBr_3_.^39^ Its crystal structure, solved at 173 K, is orthorhombic, space group *Pnma*, and it adopts a distorted 4H perovskite-like structure with face- and corner-sharing octahedra.^39^

Since temperature-dependent structural changes of IMPbBr_3_ and its optical and electrical properties are unknown, we decided to conduct comprehensive thermal, single-crystal X-ray diffraction (SCXRD), Raman, optical, PL, and dielectric studies of this perovskite. We will show that IMPbBr_3_ undergoes two structural phase transitions below room temperature (RT) and that this compound exhibits intense red PL as well as switchable dielectric properties. We also successfully grew large crystals of previously unreported IMI_2_PbBr_4_ and IM_3_PbBr_5_ with unique crystal structures, which undergo structural phase transitions and exhibit broadband PL. Furthermore, IM_3_PbBr_5_ is a rare example of a 1D perovskite exhibiting polar order at low temperatures.

## Experimental Details

### Synthesis

All reagents (PbBr_2_ 98%, imidazolium 99%, HBr 48 wt % in H_2_O) used for the synthesis were commercially purchased from Sigma-Aldrich and used without further purification. To grow single crystals of IMPbBr_3_, a solution was prepared by dissolving 5 mmol of imidazole and 5 mmol of PbBr_2_ in hydrobromic acid. The clear solution was obtained after stirring for 20 min and then left for crystallization at room temperature (RT). The same method was used to crystalize IM_2_PbBr_4_ and IM_3_PbBr_5_ but with imidazolium/PbBr_2_ ratios of 2:1 and 4:1, respectively. The crystals were harvested after one week, decantated, and then dried at RT (Figure S1). The comparison of their powder X-ray diffraction (XRD) patterns with the calculated ones based on the single-crystal data attests the phase purity of bulk samples (Figure S2).

### X-ray Powder Diffraction

Powder XRD patterns were measured in the reflection mode on an X’Pert PRO X-ray diffraction system equipped with a PIXcel ultrafast line detector and Soller slits for Cu Kα_1_ radiation (λ = 1.54056 Å).

### Differential Scanning Calorimetry (DSC)

Heat flow was measured using a Mettler Toledo DSC-1 calorimeter with a high resolution of 0.4 μW. The cooling and heating speed rate was 5 K min^–1^. Sample weights were 29.0, 31.3, and 26.8 mg for IMPbBr_3_, IM_2_PbBr_4_, and IM_3_PbBr_5_, respectively. The excess heat capacity associated with the phase transition was evaluated by subtracting from the data the baseline representing the variation in the absence of the phase transitions.

### Single-Crystal X-ray Diffraction

Single-crystal X-ray diffraction (SCXRD) experiments were carried out using an Xcalibur four-circle diffractometer (Oxford Diffraction) with an Atlas CCD detector and graphite-monochromated Mo Kα radiation. Absorption was corrected by multiscan methods using CrysAlis PRO 1.171.41.93a (Rigaku Oxford Diffraction, 2020). Empirical absorption correction using spherical harmonics, implemented in the SCALE3 ABSPACK scaling algorithm, was applied. Crystal structures were solved in Olex2 1.5^40^ using SHELXT^41^ and refined with SHELXL.^42^ For all structures measured at 295 K or less, H-atom parameters were constrained, while for measurements at 415 K (IM_2_PbBr_4_) and 400 K (IM_3_PbBr_5_), H atoms were not inserted due to the dynamic disorder of IM^+^. The main experimental details for all of the reported compounds are shown in Tables S1–S3, together with selected geometric (Tables S4–S6) and hydrogen-bonding (Tables S8–S9) parameters. The concise structure details, *i.e*., symmetry, unit cell parameters, and refinement factors, are as follows.

IMPbBr_3_ (**I**, 295 K): hexagonal, *P*6_3_/*mmc*, *a* = *b* = 9.0871(5) Å, *c* = 13.8501(7) Å, *V* = 990.45(12) Å^3^, *Z* = 4, *R*_1_ = 0.03, w**R**_2_ = 0.06, *S* = 1.08; (**II**, 220 K): orthorhombic, *P*2_1_2_1_2_1_, *a* = 9.0432(8) Å, *b* = 13.7661(7) Å, *c* = 31.053(3) Å, *V* = 3865.8(5) Å^3^, *Z* = 16, *R*_1_ = 0.13, w**R**_2_ = 0.38, *S* = 1.05; (**III**, 190 K): orthorhombic, *Pna*2_1_, *a* = 14.2042(3) Å, *b* = 8.8719(2) Å, *c* = 14.9262(2), *V* = 1880.97(6) Å^3^, *Z* = 8, *R*_1_ = 0.03, w**R**_2_ = 0.06, *S* = 1.09.

IM_2_PbBr_4_ (**I**, 415 K): orthorhombic, *Cmmm*, *a* = 9.2682(8) Å, *b* = 27.049(4) Å, *c* = 6.1301(5) Å, *V* = 1536.8(3) Å^3^, *Z* = 4, *R*_1_ = 0.05, w**R**_2_ = 0.12, *S* = 1.05; (**II**, 295 K): triclinic, *P*1̅, *a* = 6.0676(2) Å, *b* = 9.3485(4) Å, *c* = 13.7370(7) Å, α = 74.336(4)°, β = 87.025(3)°, γ = 88.612(3)°, *V* = 749.21(6) Å^3^, *Z* = 2, *R*_1_ = 0.03, w**R**_2_ = 0.08, *S* = 1.08; (**III**, 150 K): triclinic, *P*1̅, *a* = 6.0086(2) Å, *b* = 9.4176(3) Å, *c* = 13.4597(5) Å, α = 73.804(3)°, β = 86.516(2)°, γ = 87.157(2)°, *V* = 729.64(4) Å^3^, *Z* = 2, *R*_1_ = 0.02, w**R**_2_ = 0.06, *S* = 1.07.

IM_3_PbBr_5_ (**I**, 400 K): orthorhombic, *Cmmm*, *a* = 9.2708(18) Å, *b* = 18.346(4) Å, *c* = 6.1547(8) Å, *V* = 1046.8(3) Å^3^, *Z* = 2, *R*_1_ = 0.05, w**R**_2_ = 0.13, *S* = 1.03; (**II**, 295 K): triclinic, *P*1̅, *a* = 6.0690(2) Å, *b* = 9.3749(5) Å, *c* = 9.7338(6) Å, α = 66.98(1)°, β = 86.87(1)°, γ = 88.62(1)°, *V* = 508.97(5) Å^3^, *Z* = 1, *R*_1_ = 0.03, w**R**_2_ = 0.07, *S* = 1.04; (**III**, 100 K): triclinic, *P*1, *a* = 5.9724(3) Å, *b* = 9.3996(4) Å, *c* = 9.4745(5) Å, α = 67.42(1)°, β = 85.47(1)°, γ = 86.93(1)°, *V* = 489.42(4) Å^3^, *Z* = 1, *R*_1_ = 0.03, w**R**_2_ = 0.05, *S* = 1.05.

### Raman Measurements

Temperature-dependent Raman spectra were measured using a Renishaw inVia Raman spectrometer, equipped with a confocal DM2500 Leica optical microscope, a thermoelectrically cooled CCD as a detector, and a diode laser operating at 830 nm. The temperature was controlled using a THMS600 stage, and the spectral resolution was 2 cm^–1^.

### Dielectric Studies

The dielectric measurements were performed using a Novocontrol Alpha impedance analyzer. The temperature was controlled by the Novo-Control Quattro system using a nitrogen gas cryostat. All dielectric measurements were taken every 1 K during the cooling cycle. Low temperatures were achieved using liquid nitrogen. The gas flow cryostat ensures temperature stability better than 0.1 K. Since the obtained single crystals were not large enough to perform single-crystal dielectric measurements, pellets made of well-dried samples were measured instead. The silver paste was deposited on the pellet surface to ensure good electrical contact. The AC voltage with amplitude 1 V and frequency in the range 1 Hz to 1 MHz was applied across the sample.

### Absorption and Photoluminescence Studies

RT absorption spectra of the powdered samples were measured using a Varian Cary 5E UV–vis–near-infrared (NIR) spectrophotometer. Emission spectra at various temperatures under 266 or 375 nm excitation from a diode laser were measured with the Hamamatsu photonic multichannel analyzer PMA-12 equipped with a BT-CCD linear image sensor. The temperature of the single-crystal sample was controlled using a Linkam THMS600 heating/freezing stage. The quantum efficiency was measured on a Hamamatsu Absolute PL quantum yields (PLQYs) measurement system C9920-02G.

### SHG

Temperature-resolved SHG studies were performed using a laser system employing a wavelength-tunable Topaz Prime vis–NIR optical parametric amplifier (OPA) pumped by the Coherent Astrella Ti:Sapphire regenerative amplifier providing femtosecond laser pulses (800 nm, 75 fs) at a 1 kHz repetition rate. The output of OPA was set to 1400 (IMPbBr_3_) or 1500 nm (IM_2_PbBr_4_ and IM_3_PbBr_5_) and was used unfocused. Laser fluence values of samples were equal to 0.28 and 0.32 mJ cm^–2^ at 1400 and 1500 nm, respectively.

The single crystals of IMPbBr_3_, IMI_2_PbBr_4_, and IMI_3_PbBr_5_ were crushed with a spatula and sieved through an Aldrich mini-sieve set, collecting a microcrystal size fraction of 125–177 μm. Next, size-graded samples were fixed in between microscope glass slides to form tightly packed layers, sealed, and mounted to the horizontally aligned sample holder. No refractive index matching oil was used. The employed measurement setup operates in the reflection mode. Specifically, the laser beam delivered from OPA was directed onto the sample at 45° to its surface. Emission-collecting optics consisted of a ⌀25.0 mm plano-convex lens of focal length 25.4 mm mounted to the 400 μm 0.22 NA glass optical fiber and was placed along the normal to the sample surface. The distance between the collection lens and the sample was equal to 30 mm. The spectra of the nonlinear optical responses were recorded by an Ocean Optics Flame T fiber-coupled CCD spectrograph with a 200 μm entrance slit. Scattered pumping radiation was suppressed with the use of a Thorlabs 800 nm shortpass dielectric filter (FESH0800). Temperature control of the sample was performed (d*T*/d*t* = 5 K min^–1^) using a Linkam LTS420 heating/freezing stage. Temperature stability was equal to 0.1 K.

## Results and Discussion

### DSC

**Figure 1:**
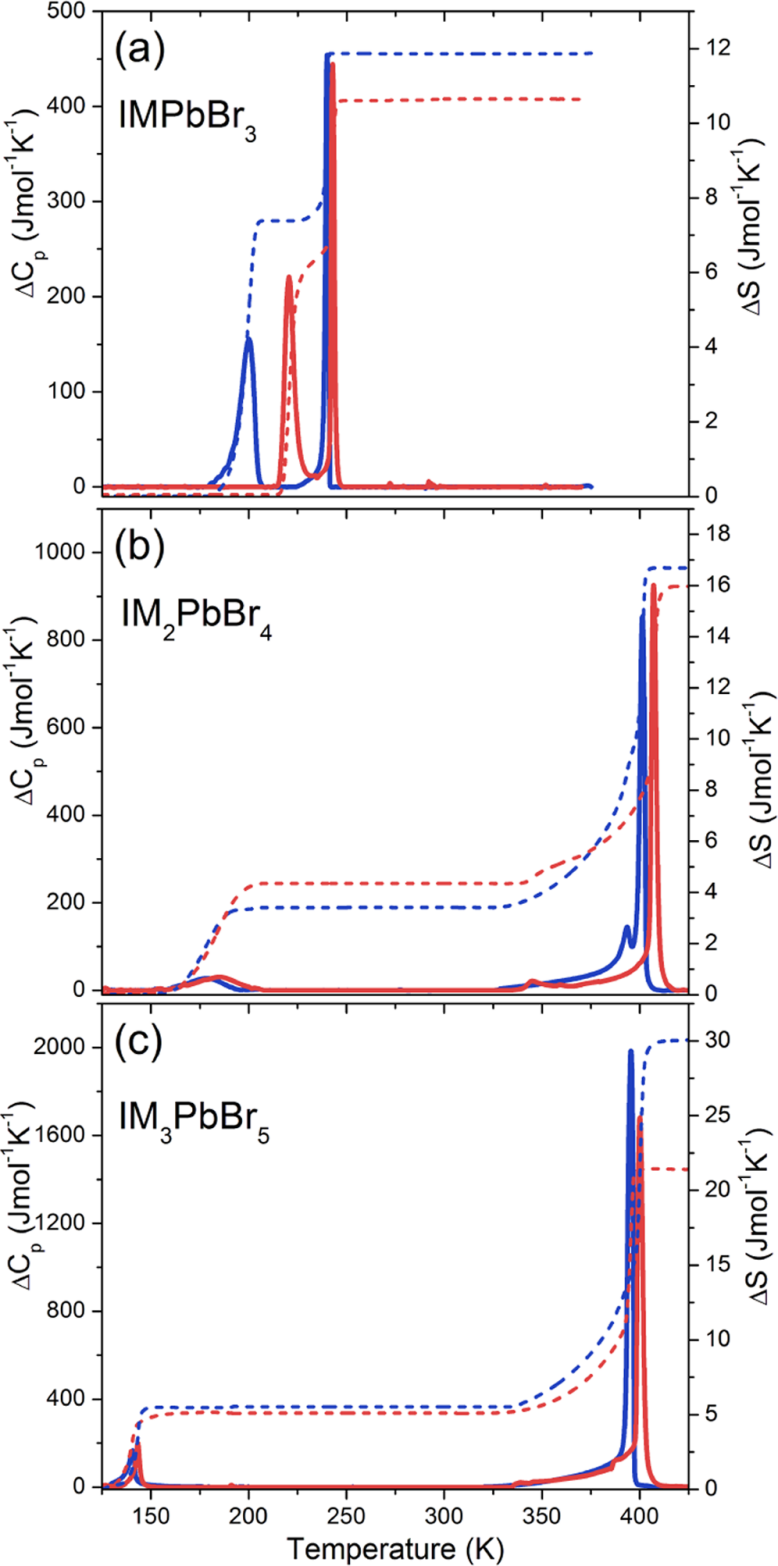
Temperature dependence of Δ*C*_p_ (solid lines) and Δ*S* (dash lines) related to (a) IMPbBr_3_, (b) IM_2_PbBr_4_, and (c) IMI_3_PbBr_5_; the red color stands for heating and blue for cooling.

The DSC measurements for IMPbBr_3_ show two anomalies at *T*_1_ = 243/240 and *T*_2_ = 221/200 K upon heating/cooling, confirming the existence of two reversible phase transitions (Figures 1a and S3). The symmetric shapes of these anomalies indicate that these phase transitions are of the first-order type. The associated changes in entropy (Δ*S*) and enthalpy (Δ*H*) were estimated to be ∼4.47 J mol^–1^ K^–1^ and ∼1.05 kJ mol^–1^ for the phase transition at *T*_1_ and ∼7.41 J mol^–1^ K^–1^ and ∼1.43 kJ mol^–1^ for the phase transition at *T*_2_. Based on the Boltzmann equation, Δ*S* = *R* ln(*N*), where *R* is the gas constant and *N* denotes the ratio of the number of distinguishable orientations, the value of *N* was calculated as 1.71 (2.43) for the phase transition at *T*_1_ (*T*_2_). These values indicate that the phase transitions are of an order–disorder type.

Two anomalies are also observed for IM_2_PbBr_4_ at *T*_1_ = 407/401 and *T*_2_ = 185/178 K (Figure 1b). The calculated values of Δ*S*, Δ*H*, and *N* are ∼12.44 J mol^–1^ K^–1^, ∼4.18 kJ mol^–1^, and 4.46 for the phase transition at *T*_1_ and ∼3.88 J mol^–1^ K^–1^, ∼0.69 kJ mol^–1^, and *N* = 1.60 for the phase transition at *T*_2_, respectively. For IM_3_PbBr_5_, the phase transitions are observed at *T*_1_ = 400/395 and *T*_2_ = 143/141 K (Figures 1c and S3). The estimated average values of Δ*S*, Δ*H*, and *N* are ∼20.41 J mol^–1^, ∼6.72 kJ mol^–1^ K^–1^, and 11.59 for the phase transition at *T*_1_ and ∼5.31 J mol^–1^ K^–1^, ∼1.04 J mol^–1^, and 1.89 for the phase transition at *T*_2_, respectively. As can be noticed, the *N* values are very large for IM_2_PbBr_4_ and IM_3_PbBr_5_ at *T*_1_, indicating a very pronounced disorder of the HT phases I, which decreases significantly in the intermediate phases **II**. The anomalies at *T*_2_ are much weaker, implying either weaker order–disorder contribution or the displacive character of the phase transitions to phases **III**.

### Single-Crystal X-ray Diffraction

#### Crystal Structure and Phase Transitions in the IMPbBr_3_ 4H-Hexagonal Perovskite

The crystal structure of IMPbBr_3_, solved at 173 K, has already been reported.^39^ It is a 3D derivative of the perovskite structure built of face-sharing PbBr_6_ octahedra, which additionally share the free corners to form a three-dimensional network. This so-called 4H-hexagonal polytype develops channels propagating along the *c*-direction, which are occupied by protonated amines. Elliot et al. reported the low-temperature (LT) structure of the orthorhombic *Pnma* symmetry.^39^ Due to the fact that this compound exhibits two phase transitions at 200 and 240 K, we decided to follow the thermal evolution of its structure. Below, we summarize the results.

**Figure 2:**
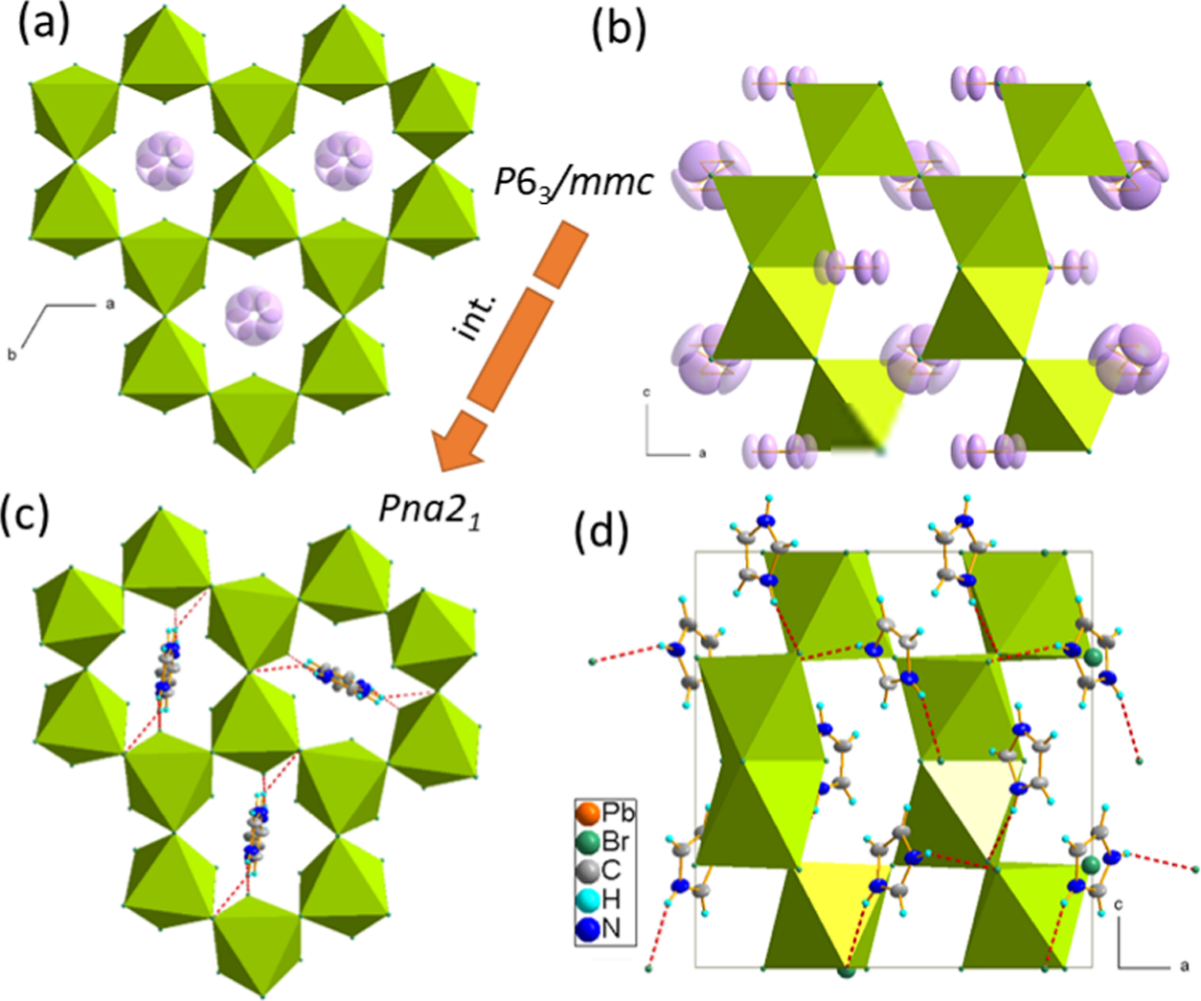
(a, b) Crystal structure of IMPbBr_3_ at RT phase I; the disordered C/N positions are drawn in pink. (c, b) Ordered LT phase **III**; IM^+^ are anchored *via* N–H···Br HBs (dashed red lines).

At RT, IMPbBr_3_ crystallizes in the hexagonal *P*6_3_/*mmc* symmetry, which is characteristic of 4H-hexagonal perovskites.^39,43^ This high, hexagonal symmetry implies the disorder of IM^+^ amines, which is illustrated in Figure 2a,b. At 295 K, all imidazolium counterions are heavily disordered; thus, it was impossible to assign the positions of N and C atoms from the diffused electron density. However, two inequivalent sites for cations could be distinguished. The first one permits free rotations of IM^+^ in all directions, and the second one allows only in-plane movements. The lack of hydrogen bonds (HBs) leaves the PbBr_6_ octahedra almost undisturbed, with negligible octahedral distortion and Pb–Br distances changing between 3.01 and 3.02 Å; see Table 1.

**Table 1:** Selected Geometric Parameters for IMPbBr_3_, IM_2_PbBr_4_, and IM_3_PbBr_5_ in All Phases^a^

compound	phase	space group	*T* (K)	Δ*d* × 10^4^	σ^2^ (deg^2^)	Br–Pb–Br_cis_ (deg)	Br–Pb–Br_trans_ (deg)	Pb–Br_av_ (Å)
IMPbBr_3_	**I**	*P*6_3_/*mmc*	295	0.05	38.2	81–98	168	3.015
	**II**	*P*2_1_2_1_2_1_	220	2.6	45.0	80–101	166–170	3.000
	**III**	*Pna*2_1_	190	1.9	36.5	81–100	172–174	3.024
IM_2_PbBr_4_	**I**	*Cmmm*	415	3.4	2.9	85–92	177–179	3.031
	**II**	*P*1̅	295	2.2	3.0	86–92	177–179	3.025
	**III**	*P*1̅	150	4.4	7.1	86–96	172–178	3.016
IM_3_PbBr_5_	**I**	*Cmmm*	400	1.1	0.4	89–91	180	3.033
	**II**	*P*1̅	295	0.1	2.0	88–92	180	3.022
	**III**	*P*1	100	1.2	10.7	85–95	170–178	3.008

a Δ*d*, bond-length distortion; σ^2^, octahedral angle variance; av, average distance in Å.

The phase transition at 240 K leads to symmetry reduction to the orthorhombic structure, with the lattice parameters being the 2-fold superstructure of the ortho-hexagonal C-centered phase with the following relationships between the lattice parameters: *a*_o_ = *a*_h_, *b*_o_ = *c*_h_, *c*_o_ = 2(2*b*_h_ – *a*_h_). Due to the twinning, the only reasonable model of the structure could be solved in the *P*2_1_2_1_2_1_ symmetry, which has not been evidenced in the SHG results (see the next paragraph). However, still, this nonperfect refinement may shed light on the atom rearrangement in the intermediate phase. Figure S4 shows the asymmetric unit, which consists of four independent lead ions, 12 Br^–^ ligands, and four ordered IM^+^ counterions. The large displacement parameters may originate from the poor quality of the twinned data set, as well as from the nonresolved disorder associated with small-angle librations of the amines. As the DSC analysis implies the order–disorder mechanism of the first phase transition, the presented model seems reasonable. Additionally, the chiral *P*2_1_2_1_2_1_ intermediate phase has been found in two DMA 4H perovskite derivatives (DMA^+^ = dimethylammonium cation).^43^

With further cooling, the second phase transition occurs, and it is associated with the translational and space group symmetry change. The volume of the unit cell is reduced twice from 3866 down to 1881 Å^3^, and the symmetry is noncentrosymmetric *Pna*2_1_ (which is confirmed by the SHG). The phase transition is triggered by the alternating arrangement of the IM^+^, which couples to the antiphase rotation of the octahedra around the *b*-axis. Although the view of the structure, displayed in Figure 2c,d, very much looks like the reported *Pnma* phase,^39^ the lack of the symmetry center has an irrefutable influence on the physical properties of this phase. The dipoles assigned to the IM^+^ cations give rise to uncompensated spontaneous polarization. Examining the octahedral distortions, collected in Table 1, we may notice a rather insignificant influence of N–H···Br HBs (which in this phase form a complete N–H···Br bonded network) on bromine displacements. Thus, a negligible influence of the phase transitions may be expected on electronic and optical properties.

#### Crystal Structure and Phase Transitions in IM_2_PbBr_4_

**Figure 3:**
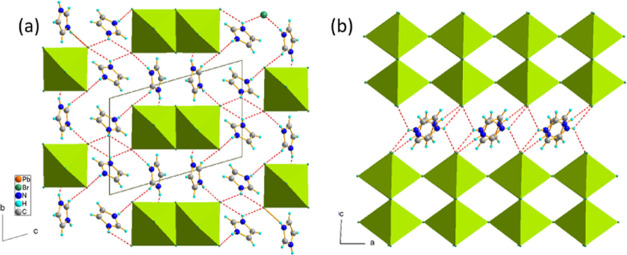
(a) Crystal structure of IM_2_PbBr_4_ consists of double chains of PbBr_6_ octahedra extending along the *a*-direction and IM^+^ interacting with the inorganic part *via* N–H···Br HBs (red dashed lines). (b) Inorganic chains are built of pairs of edge-sharing octahedra, which are connected through corners. The picture shows phase **III**, at 150 K, with established HBs.

IM_2_PbBr_4_ crystallizes in the triclinic *P*1̅ symmetry and possesses two additional polymorphic forms depending on temperature, *i.e*., a disordered, high-temperature (HT) orthorhombic *Cmmm* phase above 400 K and another triclinic *P*1̅ isomorphic phase below 178 K. The lead bromine substructure is similar to that found in C_4_N_2_H_14_PbBr_4_.^44^ In both compounds, the 1D inorganic part consists of [PbBr_4_^2–^]_∞_ double chains which are separated by protonated amines. The chains are built of edge-sharing bioctahedra, which in turn share apical bromines and form a polymeric structure expanding along the *a*-direction; see Figure 3a,b. The edge-sharing assembly is not a typical octahedral connection in 2D and 3D metal halide perovskites; however, it is present in a number of low-dimensional (0D or 1D) lead halides.^31,45,46^

**Figure 4:**
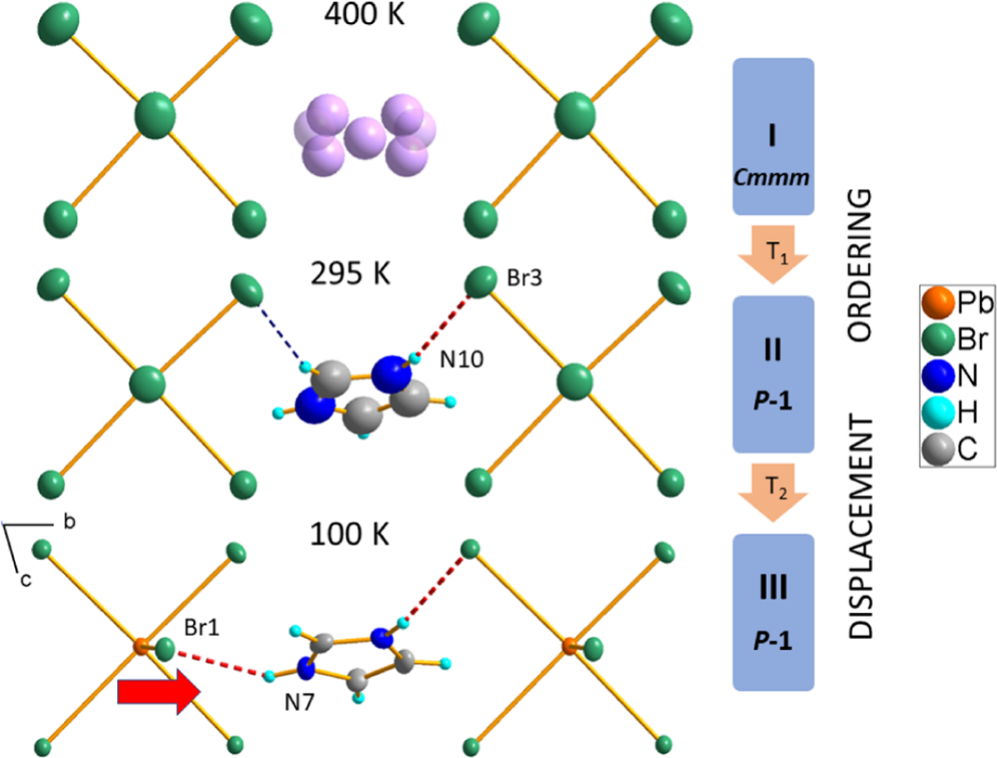
Mechanism of phase transitions in IM_2_PbBr_4_. The disordered C/N positions are drawn in pink; the thermal ellipsoid is drawn at the 50% probability level.

The phase transitions appear as a result of the interplay between the temperature-activated rotations of IM^+^, N–H···Br hydrogen bonding, and octahedral distortions of PbBr_6_ units. The HT orthorhombic phase is characterized by disordered IM^+^ cations, which may freely perform in-plane rotations. Symmetry reduction at 401 K to the triclinic, *P*1̅ arises due to the ordering of IM^+^. In this phase, the cations are anchored *via* weak N–H···Br and C–H···Br HBs; however, the hydrogen-bonded network is not complete, some NH groups do not have the hydrogen acceptor in this phase. Moreover, quite large displacement parameters of all atoms, as well as insignificant changes in octahedral distortion parameters (see Table 1), imply still weak interaction of the cations with the inorganic part of the structure. The second, isostructural phase transition at *T*_2_ = 178 K is associated with the rearrangement of the packing due to the stabilization and establishment of a complete supramolecular assembly *via* N–H···Br HBs. Figure 4 shows the mechanisms of the phase transitions in IM_2_PbBr_4_. The isostructural transformation at **II** to **III** PT is of a displacive type. A new N7–H···Br1 bond is created between IM^+^ and Br1 acting as a linkage between the bioctahedral units. As a result, the Pb–Br1 distance increases from 3.050(1) Å in **II** up to 3.070(1) Å in **III**, whereas the Pb1–Br1–Pb1 angle decreases from 178 to 172°. The unusual elongation of a bond at LT is also observed for Pb1–Br3 (from 2.994(1) Å up to 3.058(1) Å) being a product of the increase in the strength of N10–H···Br3 HB (the donor-to-acceptor distance decreases from 3.56(1) Å in **II** to 3.365(50) Å in **III**). As a result of changes in the configuration of HBs, there is a continuous increase in the shortest distance between the chains, which refers to the *b*-lattice parameter in **II** and **III.** Thus, the negative thermal expansion develops in the LT phases of IM_2_PbBr_4_ along the *b*-direction.

#### Crystal Structure and Phase Transitions in IM_3_PbBr_5_

The last polymorph IM_3_PbBr_5_ is a representative of the 1D perovskites built on [PbBr_5_]*_n_*^3–^ chains arranged from the single, corner-sharing PbBr_6_ octahedra. The motif of linear chains of corner-sharing PbX_6_ octahedra is quite rare for A_3_PbX_5_ compounds. A few examples may be found for A_3_PbI_5_ crystallizing with guanidinium,^47^ iodoformamidinium,^48^ protonated thiourea,^49^ mixed methylammonium/dimethyl sulfoxide (DMSO),^50^ A_3_PbCl_5_ representatives with protonated methyltiourea,^51^ and melamine.^52^ To the best of our knowledge, IM_3_PbBr_5_ is the first example of lead bromine with the linear chains of corner-sharing PbBr_6_ octahedra.

**Figure 5:**
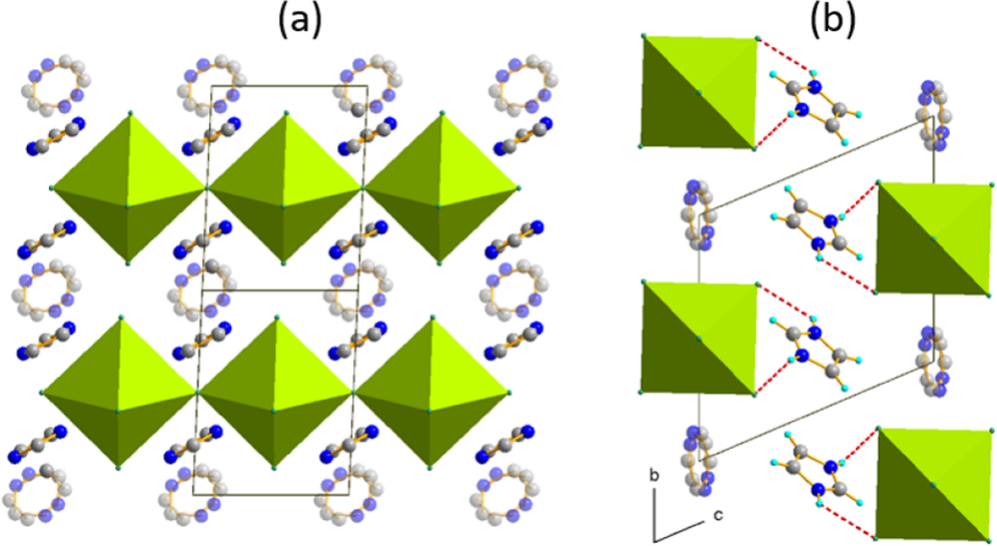
(a) Crystal structure of Im_3_PbBr_5_ at RT, projected along the (011) direction, phase **II**, triclinic *P*1̅. Corner-sharing octahedra form [PbBr_5_]*_n_*^3–^ chains extending along the *a*-direction. (b) Organic substructure is partially ordered and interacts *via* N–H···Br HBs with the chains. The transparent atoms are from disordered IM^+^.

The RT structure is shown in Figure 5. The lead halide chains are separated by two sets of inequivalent IM^+^ moieties, stacking along the *b*-direction and along the chains (in the *a*-direction). As in the previous compounds, three phases induced by temperature can be distinguished in this material. In the HT phase of the orthorhombic *Cmmm* symmetry, which is stabilized above 395 K, all IM^+^ cations are disordered and perform in-plane and out-of-plane rotations. The transition to the RT phase is triggered by the reduction of the degrees of freedom of IM^+^, which are placed in the stacks along the triclinic *b*-direction. A temporary hydrogen-bonded network is formed, in which each ordered cation is hydrogen-bonded to one inorganic chain. The second phase transformation is driven by the further IM^+^ ordering and rearrangement of the existing HBs. In **III**, which is triclinic *P*1, the complete 3D supramolecular structure is formed, as each IM^+^ interacts with two neighboring chains. Figure S5 shows thermally induced changes in the crystal structure. The lack of the symmetry center implies polar properties of this phase originating from noncompensated dipoles of IM^+^.

The strong N–H···Br HBs influence the packing of the crystal structure. The shortest distance between the chains, which actually refers to the *b*-lattice parameter, increases with the temperature lowering from 9.27 Å (**I**) to 9.375 Å (**II**) and finally to 9.400 Å (**III**), implying negative thermal expansion along this direction.

In all IM_3_PbBr_5_ polymorphs presented here, the octahedral distortion parameters (Δ*d* = 0.1–1.2, σ^2^ = 0.4–10.7 deg^2^, Table 1) seem to be negligible compared to 2D (MHy_2_PbBr_4_, Δ*d* = 2.1–4.2, σ^2^ = 11.3–34.9 deg^2^) and 3D (MHyPbBr_3–*x*_Cl*_x_*, Δ*d* = 0.55–14.6, σ^2^ = 14–314 deg^2^) perovskites reported by us earlier.^10,11,22^

### NLO Studies

The following section is devoted to the NLO spectroscopic verification of whether phases **II** and **III** of IM_2_PbBr_4_ and IM_3_PbBr_5_ feature acentric structural order or not. The screening of SHG activity was performed using 1500 nm ultrafast laser pulses for all crystal phases that were detected using DSC and crystallography (*vide supra*). Indeed, in our SHG studies of lead halide perovskites, we generally employ long-wavelength femtosecond laser excitations (1300–1500 nm), for several reasons. One is that lead halide perovskites, with their optical band gaps mainly located in the visible spectral range, are very good two-photon absorbers at corresponding two-photon resonances at doubled wavelengths;^17^ accordingly, the irradiation of perovskite samples with, *e.g*., 800 nm laser pulses, typically results in giant two-photon excited luminescence that largely obscures the SHG response.^11,18^ The other factor is that intense excitation through resonant nonlinear absorption processes can lead to the photochemical generation of defects and associated with this modification of the optical properties such as luminescence quenching or emergence of defect-derived SHG response. Thus, to minimize convoluting phenomena, it is preferred to shift away the laser excitation deep into the NIR region, where multiphoton absorption cross sections are much lower in intensity so that the upconverted emissions are correspondingly weaker.

**Figure 6:**
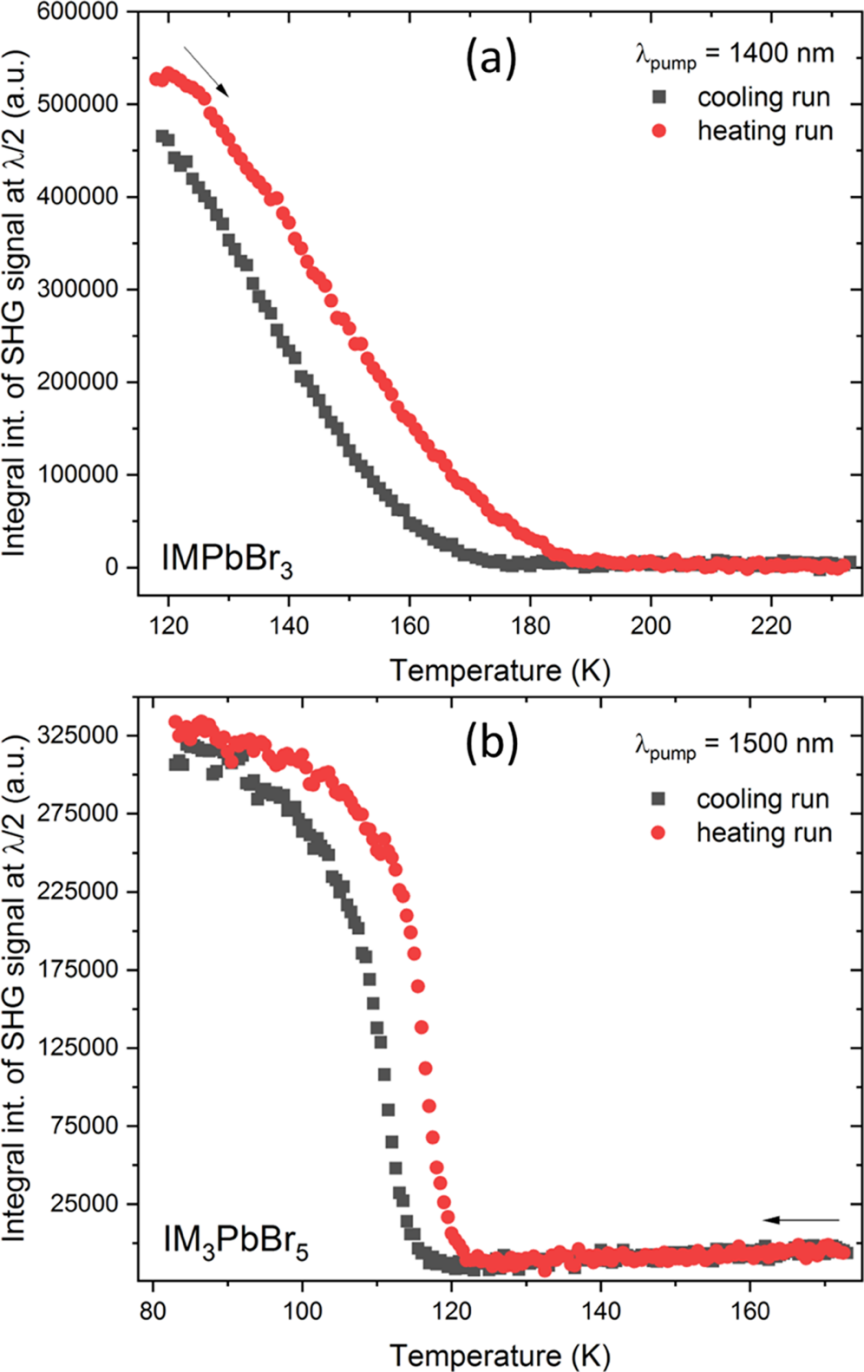
Integral intensities of SHG signals of (a) IMPbBr_3_ and (b) IM_3_PbBr_5_ for cooling (black squares) and heating runs (red circles) plotted as a function of temperature.

Screening of the SHG activity for IMPbBr_3_ and IM_3_PbBr_5_ has been realized through the temperature-resolved irradiation experiments of the size-graded samples with 1400 and 1500 nm femtosecond laser pulses, respectively. Figure 6 displays calculated integral areas of second-harmonic responses for heating and cooling runs plotted as a function of temperature, while corresponding spectra of registered nonlinear optical responses are shown in Figure S6.

Collected results for IMPbBr_3_ demonstrate that out of three crystal phases only the LT phase **III** produces SHG. Indeed, upon cooling, the SHG signal at 700 nm could be detected as low as at 175 K and is partially overlapped with weak multiphoton excited luminescence (MPEL) (Figure S6a); upon the heating run, SHG is no longer detected at about 185 K (Figure S6b). Thus, plots of integral SHG intensities for cooling and heating runs are separated by *ca*. 10 K-wide thermal hysteresis (Figure 6a).

It is apparent that HT phase **II** of IM_3_PbBr_5_ displays broad, moderate MPEL (Figure S6c,d), but no SHG response is present. This confirms the centrosymmetric character of this crystal phase. However, one sees that upon cooling below 120 K the SHG signal at 750 nm starts to emerge and sits at the shoulder of the broad MPEL emission (Figure S6c), which also increases its intensity due to decreased thermal quenching. Overall, it is clear that only the LT phase **III** of IM_3_PbBr_5_ is noncentrosymmetric.

Under identical irradiation conditions (1500 nm), the screening of the SHG activity was also performed for IM_2_PbBr_4_. In this case, only MPEL signals of various intensities have been registered, but no trace of SHG at 750 nm could be noticed (Figure S7). Combined with crystallographic results, it led us to the conclusion that all three crystal phases of IM_2_PbBr_4_ feature centrosymmetric structures.

### Dielectric Studies

**Figure 7:**
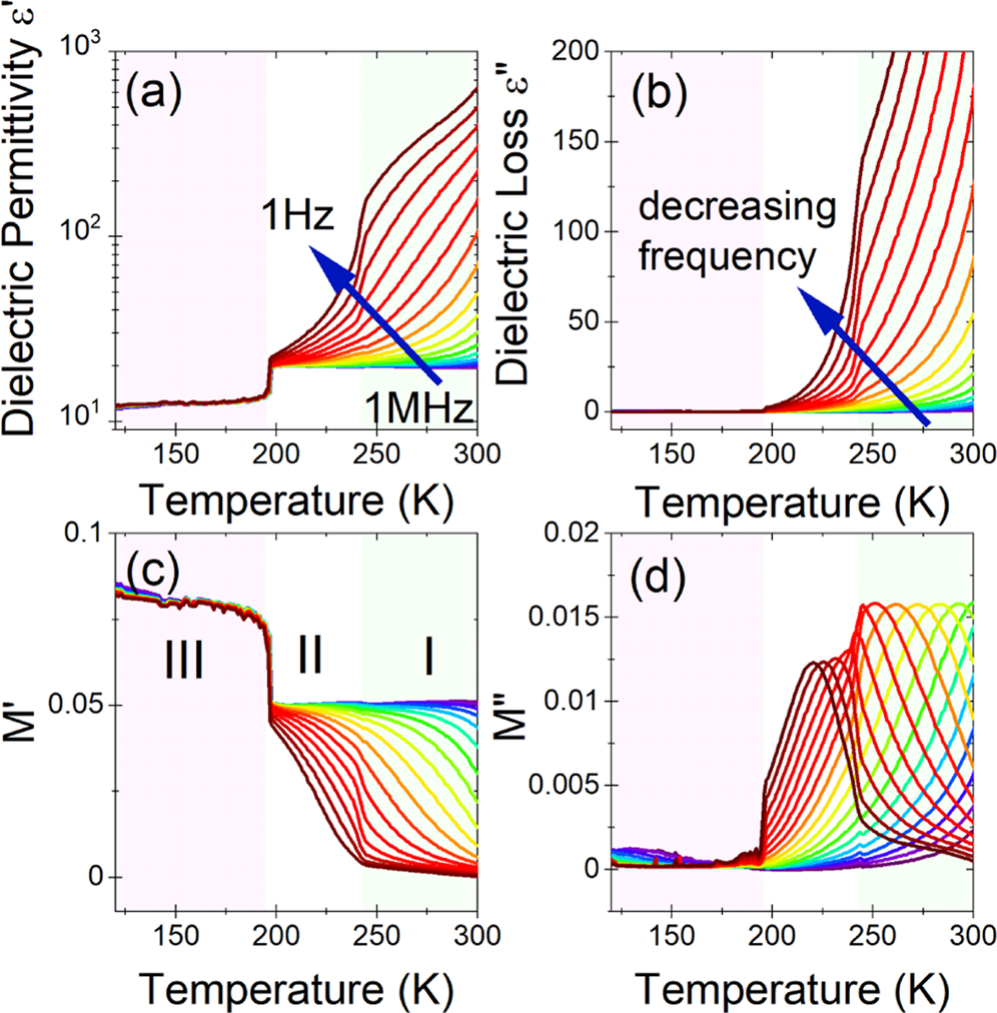
Temperature dependence of real (a) and imaginary (b) parts of dielectric permittivity of the IMPbBr_3_ sample. Complex electric modulus (M* = 1/ε*) *vs* temperature for its real (c) and imaginary (d) components. The representative curves are plotted in frequency decades between 1 Hz and 1 MHz. The marked areas correspond to different phases.

**Figure 8:**
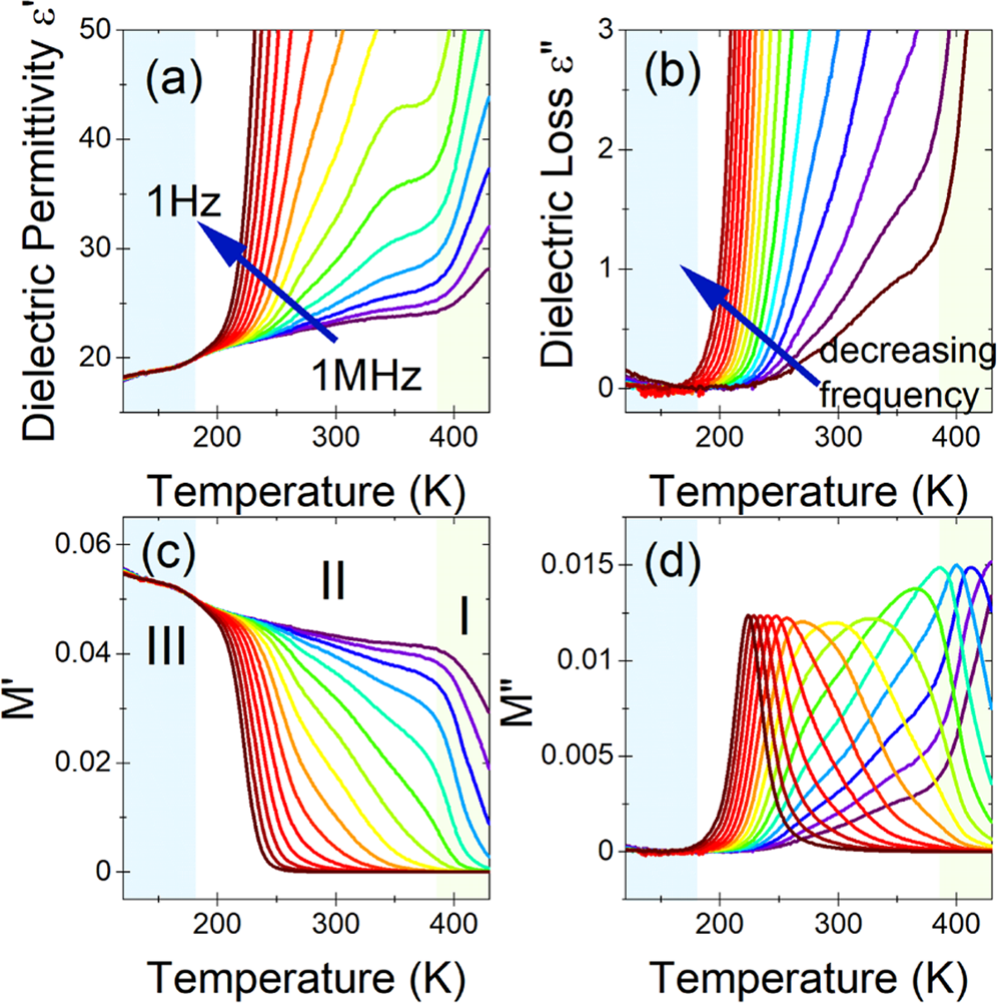
Temperature dependence of real (a) and imaginary (b) parts of dielectric permittivity of the IM_2_PbBr_4_ sample. Complex electric modulus (M* = 1/ε*) *vs* temperature for its real (c) and imaginary (d) components. The representative curves are plotted in frequency decades between 1 Hz and 1 MHz. The marked areas correspond to different phases.

**Figure 9:**
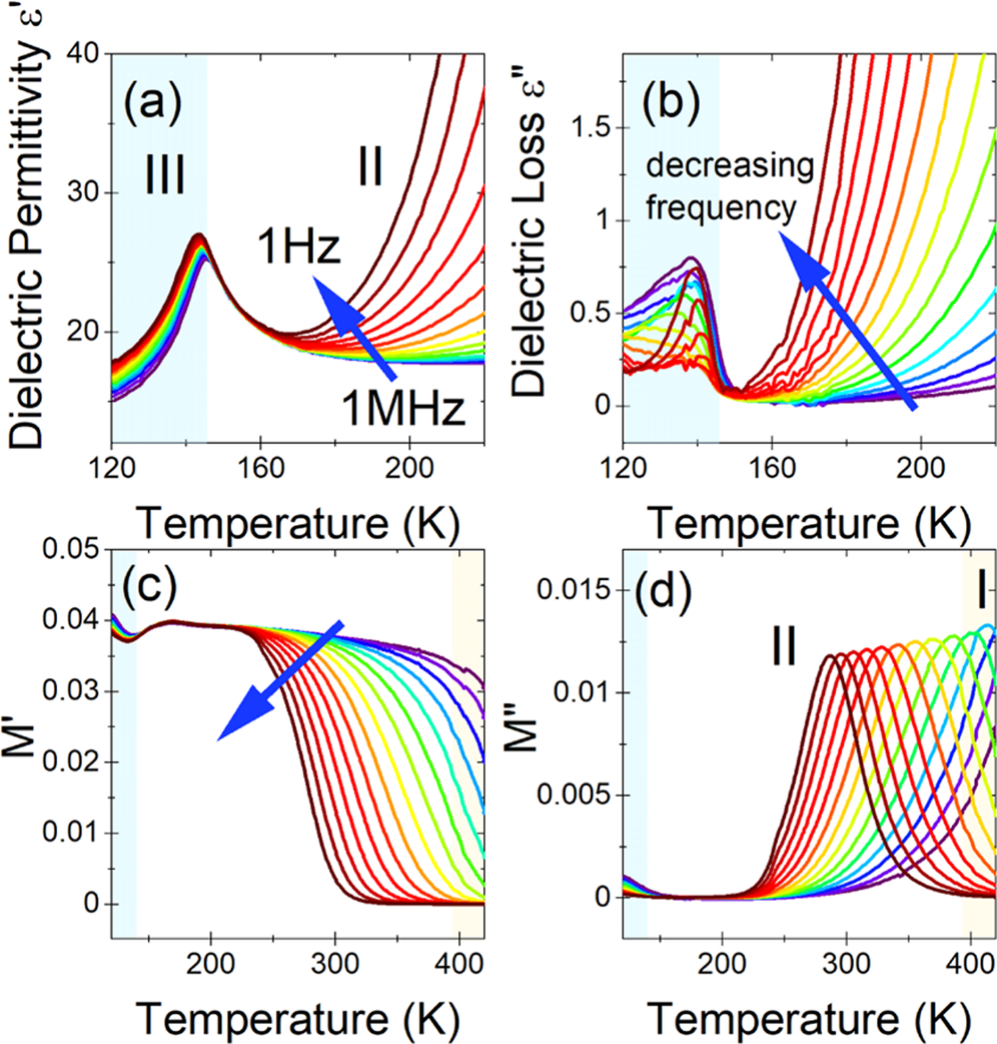
Temperature dependence of dielectric permittivity ε′ (a) and dielectric loss ε″ (b) of the IM_3_PbBr_5_ compound. Temperature of the real (c) and imaginary (d) components of the electric modulus (M* = 1/ε*) *vs* temperature. The representative curves are plotted in frequency decades between 1 Hz and 1 MHz. The marked areas correspond to different phases.

In many organic–inorganic hybrid compounds, the temperature-induced structural ordering leads to the appearance of a dielectric relaxation process.^53^ This fact motivated us to perform comprehensive temperature-dependent dielectric measurements of IMPbBr_3_, IM_2_PbBr_4_, and IMI_3_PbBr_5_. To depict the structural changes associated with the changes in the internal dipole moment, the temperature-dependent complex dielectric permittivity ε* (ε* = ε′ – iε″) spectra were collected (Figures 7a,b, 8a,b, and 9a,b). Moreover, to suppress the electrode effect, the modulus representation (M* = 1/ε*) was also used (Figures 7c,d, 8c,d, and 9c,d). For the IMPbBr_3_, the tendency of the dielectric spectra is that ε′ increases monotonically with increasing temperature at all frequencies, but anomalies confirming the structural phase transitions are also seen. In particular, the phase transition at *T*_1_ is noticed only as a subtle change in the slope, while that at *T*_2_ is accompanied by a significant leap, with a dielectric increment (Δε′) being on the order of 6 for 1 MHz (Figure 7a). Such a behavior of dielectric permittivity classifies this material as a potential switchable organic–inorganic dielectric.^54,55^ Above *T*_2_, a distinct frequency dispersion observed in the dielectric spectra indicates the onset of some thermally activated relaxation process (Figure 7a,b). This process is further enhanced above *T*_1_, suggesting that it can be attributed to increased conductivity. This assumption is additionally confirmed by the characteristic shape of the electric modulus spectra. Interestingly, the change in symmetry at *T*_1_ distinctly influences the observed relaxation process shown on M* (Figure 7c,d).

The dielectric spectra of IM_2_PbBr_4_ are similar in shape and temperature tendency to those recorded for IMPbBr_3_, but the observed changes in the phase transitions are more subtle (Figure 8a–d). Starting from the lowest temperature, the dielectric permittivity reaches values below 20 (Figure 8a). This behavior resembles that observed for IMPbBr_3_ (Figure 7a) and indicates that in both compounds the electrical polarizability at low temperatures depends only on the chemical components of the compounds and the contribution from structural dimensionality is negligible. The phase transition from the LT phase **III** to the intermediate phase **II** at *T*_2_ = 183 K affects weakly the values of complex dielectric permittivity and the electric modulus. However, above *T*_2_, a strong frequency dispersion appears, which becomes more and more pronounced with increasing temperature. As a consequence, the HT phase transition from phase **II** to phase **I** is hardly visible in the dielectric spectra. It is worth adding that the shape of the dielectric spectra suggests that the observed frequency dispersion is related to the relaxation process associated with electrical conductivity.

The dielectric response of IM_3_PbBr_5_ looks quite intriguing. At the phase transition temperature *T*_2_, observed in the dielectric data near 145 K, the observed change in dielectric permittivity has a lambda shape, reaching the value of about 25 at the peak (Figure 9a). This shape indicates temperature-induced polarizability changes, which is characteristic of ferroelectric phase transitions. However, the changes in values of ε′ are relatively small as for improper ferroelectricity. In the whole investigated temperature range, obeying two phases, strong frequency dependences are observed for both ε* and M* (Figure 9b–d). As the frequency decreases, the complex dielectric permittivity increases (Figure 9a,b). In a similar manner to the two other studied compounds, the significant frequency dispersion observed for the intermediate phase **II** and the HT phase **I** indicates the appearance of the relaxation process related to conductivity. The characteristic bell-shaped curves on the imaginary part of M* (Figure 9d) and the inverted S-shape of its real part (Figure 9c) clearly confirm the relaxation process in this compound.

To quantify the observed relaxation processes, an analysis of spectra in the frequency domain was performed. Figures S8–S10 show the temperature-dependent spectra of ε* and M* for IMPbBr_3_, IM_2_PbBr_4_, and IM_3_PbBr_5_, respectively. For the IMPbBr_3_, in the temperature range from 180 to 300 K, the frequency dependence of both the complex dielectric permittivity and the electric modulus spectra exhibit a single anomaly (depicted as a bell-shaped curve of the imaginary and steplike tendency of real parts), slightly obscured by some conductivity process. The well-depicted steplike tendency and a bell-shaped curve of real and imaginary parts, respectively, of both ε and *M*, indicate the dipolar relaxation process in this compound (Figure S8). The complex dielectric spectra for the IM_2_PbBr_4_ compound collected in the frequency domain revealed the characteristic relaxation process just on the electric modulus (Figure S9). This behavior suggests that the observed relaxation process is related to conductivity. In the case of IM_3_PbBr_5_, in the studied frequency range, two dipole relaxation processes are visible only below the phase transition temperature. The second relaxation process, observed only in the electric modulus spectra, is related to thermally induced conductivity (Figure S10).

**Figure 10:**
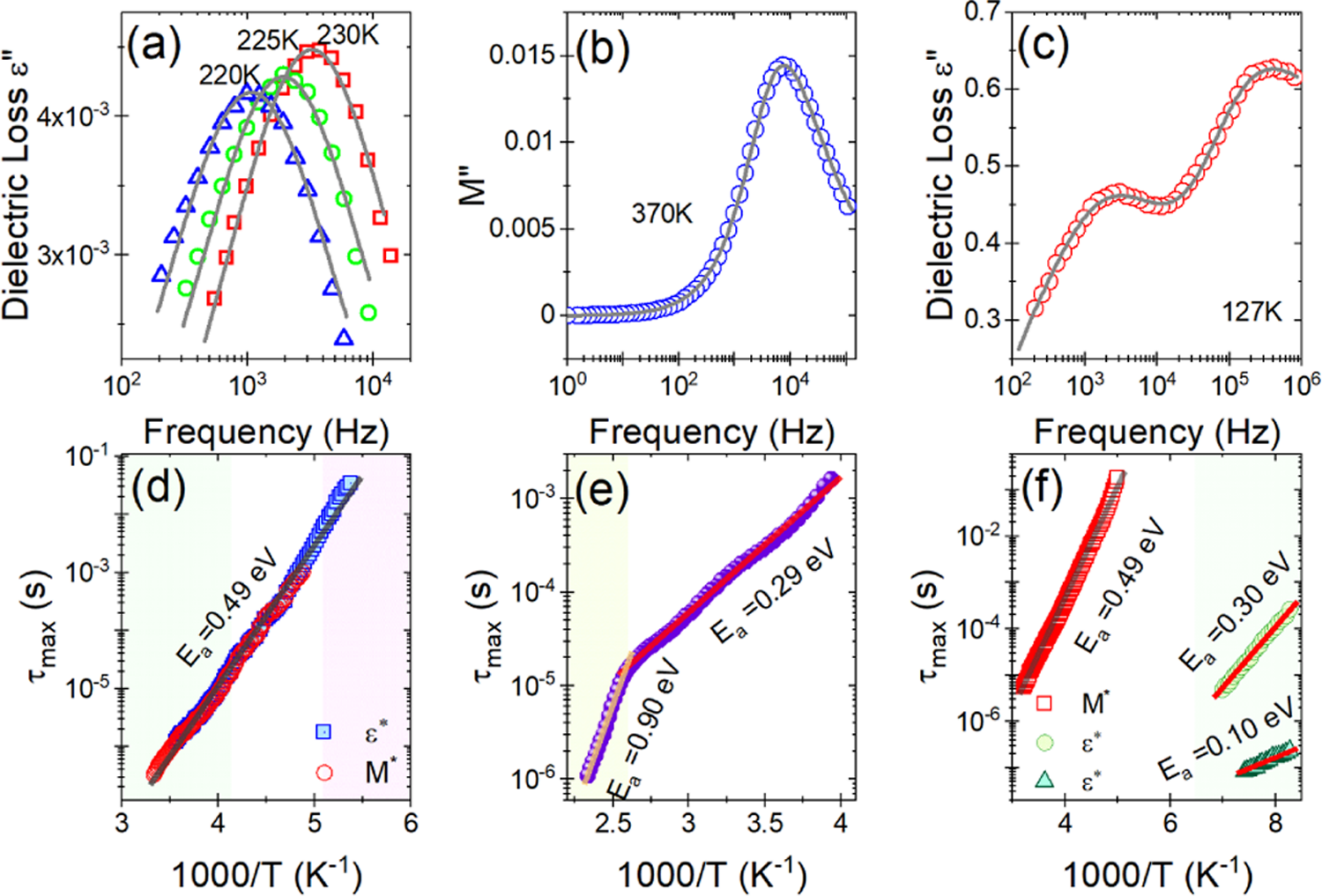
(a) Dipolar relaxation process depicted as a bell-shaped curve on dielectric loss spectra of IMPbBr_3_ for three selected temperatures, (b) conductivity process observed in electric modulus representation for IM_2_PbBr_4_, (c) frequency dependence of ε′ depicting two structural relaxation processes for IM_3_PbBr_5_. The solid lines correspond to the H–N fittings. Relaxation times of the dipolar process as a function of 1000/*T* for (d) IMPbBr_3_, (e) IM_2_PbBr_4_, and (f) IM_3_PbBr_5_.

To accurately estimate the characteristic dipolar relaxation times, in the vicinity of the peak of imaginary parts of ε and *M*, the data were parameterized using a single Havriliak–Negami (H–N) function: , where τ and Δε denote the relaxation time and strength, respectively, ε_∞_ is the high-frequency contribution, and parameters α and β describe symmetrical and asymmetrical broadening of the relaxation peak. The relaxation times were calculated from the peak maximum frequency as τ_max_ = (2π*f*_max_)^−1^. Figure 10a–c shows the type of relaxation responses and their compatibility with the H–N function using the dielectric permittivity representation detected at a selected temperature for IMPbBr_3_ and IM_3_PbBr_5_ and the electric modulus for IM_2_PbBr_4_. To depict the structural relaxation dynamics, we have determined the temperature-dependent behavior of the dielectric relaxation times obtained from both dielectric permittivity and electric modulus data τ_max_ and scaled log τ_max_ as a function of 1000/*T* (see Figure 10c–e).

In the Arrhenius plot for IMPbBr_3_ (Figure 10c), the thermal activation energy equal to 0.49 eV remains unchanged in all phases. This behavior indicates that the structural changes do not affect the dynamics of the observed dipolar relaxation process. By assigning the observed relaxation process to the movements of the disordered IM^+^ cations, it can be concluded that changes in symmetry do not affect the local surroundings of these cations. This conclusion is consistent with the structural results, which showed that the phase transitions are related to the distortion of the octahedra and the distances between the IM^+^ cations and their neighbors remain the same in the studied temperature range.

The activation energies of the conductivity process determined for the IM_2_PbBr_4_ compound are 0.9 and 0.29 eV for phases **I** and **II**, respectively (Figure 10e). The tripling of *E*_a_ when going from phase **II** to phase **I** indicates that the structural changes significantly disturb the conductivity process.

The relaxation map for IM_3_PbBr_5_ is more enriched with relaxation processes, where three different Arrhenius-type-related processes can be discerned. Two of them, visible only in the LT phase, are dipolar relaxation processes. The activation energies of these processes are, respectively, 0.3 and 0.1 eV. These two processes correspond to some specific movements of the structural elements that change their dipole moment. By assigning the observed changes to the imidazolium, it can be concluded that the movement of this cation is complex. The third HT process, which is only seen in the electric modulus spectra, is associated with the conductivity process and has an activation energy of 0.49 eV. The very value of the activation energy, however, does not make it possible to clearly state what the observed conductivity process is related to.

### Raman Scattering Studies

**Figure 11:**
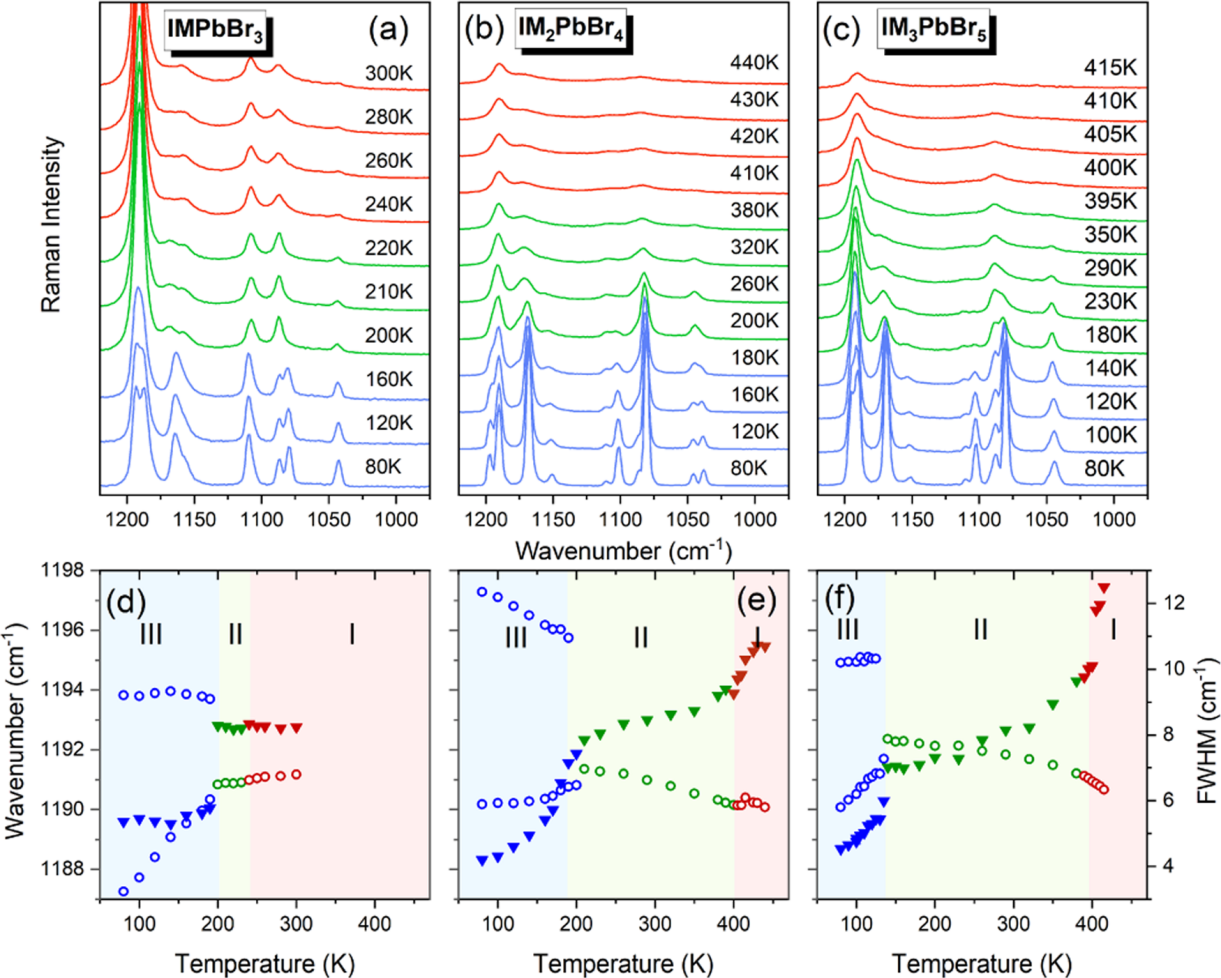
Temperature evolution of Raman spectra in the 1225–975 cm^–1^ range for (a) IMPbBr_3_ (b) IM_2_PbBr_4_, and (c) IM_3_PbBr_5_. Panels (d)–(f) present the temperature dependence of peak positions (open circles) and FWHM (full triangles) for the same compounds. The marked areas correspond to different phases.

Temperature-dependent Raman spectra of the studied compounds in the entire wavenumber range are shown in Figures S11–S13. Details for the 1225–975 cm^–1^ range, which corresponds to the internal vibrations of the imidazole ring,^56,57^ are presented in Figure 11a–c. To better monitor temperature-dependent changes in the Raman spectra, we also present temperature dependence of Raman wavenumbers and full width at half-maximum (FWHM) values (Figure 11d–f).

Raman spectra confirm that all studied compounds exhibit two phase transitions (Figure 11). They also show that the temperatures of these transitions are quite close to the values determined by DSC and that the phase transitions at *T*_2_ lead to more pronounced changes than those around *T*_1_ (Figure 11). In particular, FWHM values of the monitored bands exhibit a large increase at *T*_2_ (Figure 11), indicating that the LT phase transitions are associated with a pronounced change in the dynamic of the imidazolium ring. On a further increase in temperature, Raman bands of IM_2_PbBr_4_ and IM_3_PbBr_5_ observed near 1190 cm^–1^ exhibit a continuous increase in FWHM, followed by a moderate increase at *T*_1_ (Figure 11e,f). This behavior resembles that observed for ferroelectric MHy_2_PbBr_4_.^58^ It shows, therefore, that motions of IM^+^ cations exhibit a pronounced increase on heating and that the contribution of the IM^+^ dynamics to the phase transition mechanism at *T*_1_ is weaker compared to that at *T*_2_. IMPbBr_3_ shows different behaviors, *i.e*., for this compound, FWHM does not exhibit any clear anomaly at *T*_1_, suggesting a negligible contribution of IM^+^ dynamics to the phase transition mechanism. It is worth noting that the temperature evolution of Raman wavenumbers and FWHM shows abrupt changes at almost all phase transition temperatures for all compounds, in line with the first-order character of these transitions. The only exception is the **II** to **III** phase transition for IM_2_PbBr_4_, for which the temperature evolution of Raman bands is more gradual (Figure 11e), suggesting its second-order character.

Figures 11 and S11–S13 also show that the Raman spectrum of IMPbBr_3_ measured at 80 K is composed of a smaller number of bands than the corresponding spectra of IM_2_PbBr_4_ and IM_3_PbBr_5_. This strongly supports the crystallographic data, demonstrating triclinic symmetry of phase **III** in IM_2_PbBr_4_ and IM_3_PbBr_5_ and orthorhombic symmetry of phase **III** in IMPbBr_3_. When the temperature increases, many weak intensity bands disappear and the splitting of some Raman bands vanishes at *T*_2_ (Figures 11 and S11–S13). These changes are consistent with the increase in the crystal symmetry when going from phases **III** to phases **II**. Interestingly, the phase transitions from phases **II** to phases **I** do not lead to any apparent change in the number of Raman bands despite clear symmetry changes revealed by the X-ray diffraction method. This behavior can be most likely attributed to the fact that IM^+^ cations in phases **II** and **III** of the studied compounds have very similar structures.

### Linear Optical Properties

Diffuse reflectance spectra registered for the investigated perovskites consist of absorption in the UV range and the excitonic bands centered at 397 (3.12), 376 (3.30), and 378 (3.28) nm (eV) for IMPbBr_3_, IM_2_PbBr_4_, and IM_3_PbBr_5_, respectively (Figure S14). The energy band gaps (*E*_g_) of these compounds, calculated using the Kubelka–Munk formula, are 3.24, 3.58, and 3.52 eV, respectively (Figure S15). These band gaps are significantly larger than reported for 3D MAPbBr_3_, FAPbBr_3_, and MHyPbBr_3_ hybrid perovskites (2.18–2.48 eV).^11,59^ According to literature data, a decrease in dimensionality and change in PbBr_6_ connectivity from corner-shared to edge- or face-shared lead to an increase in the band gap and a blue shift of the excitonic absorption. For instance, band gaps near above 3 eV were reported for many 2D layered corner-shared bromide perovskites,^34,60−62^ as well as 0D and 1D analogues containing corner-, edge-, or face-shared PbBr_6_ octahedra.^31,45,46,63,64^ Thus, large band gaps of the studied compounds are consistent with their low dimensionality. Since IMPbBr_3_ is isostructural to DMAPbBr_3_,^43^ it is worth comparing band gaps and excitonic absorptions of these compounds. Using our data reported previously for DMAPbBr_3_,^65^ we estimate the excitonic absorption and the band gap of this compound as 388 nm (3.20 eV) and 3.34 eV, respectively. Accordingly, IMPbBr_3_ shows more red-shifted excitonic absorption and a narrower band gap compared to DMAPbBr_3_. Taking into account that the band gap and energy of excitonic absorption increase with increasing distortion of the inorganic network,^10,29,62,66^ the observed differences between IMPbBr_3_ and DMAPbBr_3_ indicate a smaller octahedral distortion in the former case.

**Figure 12:**
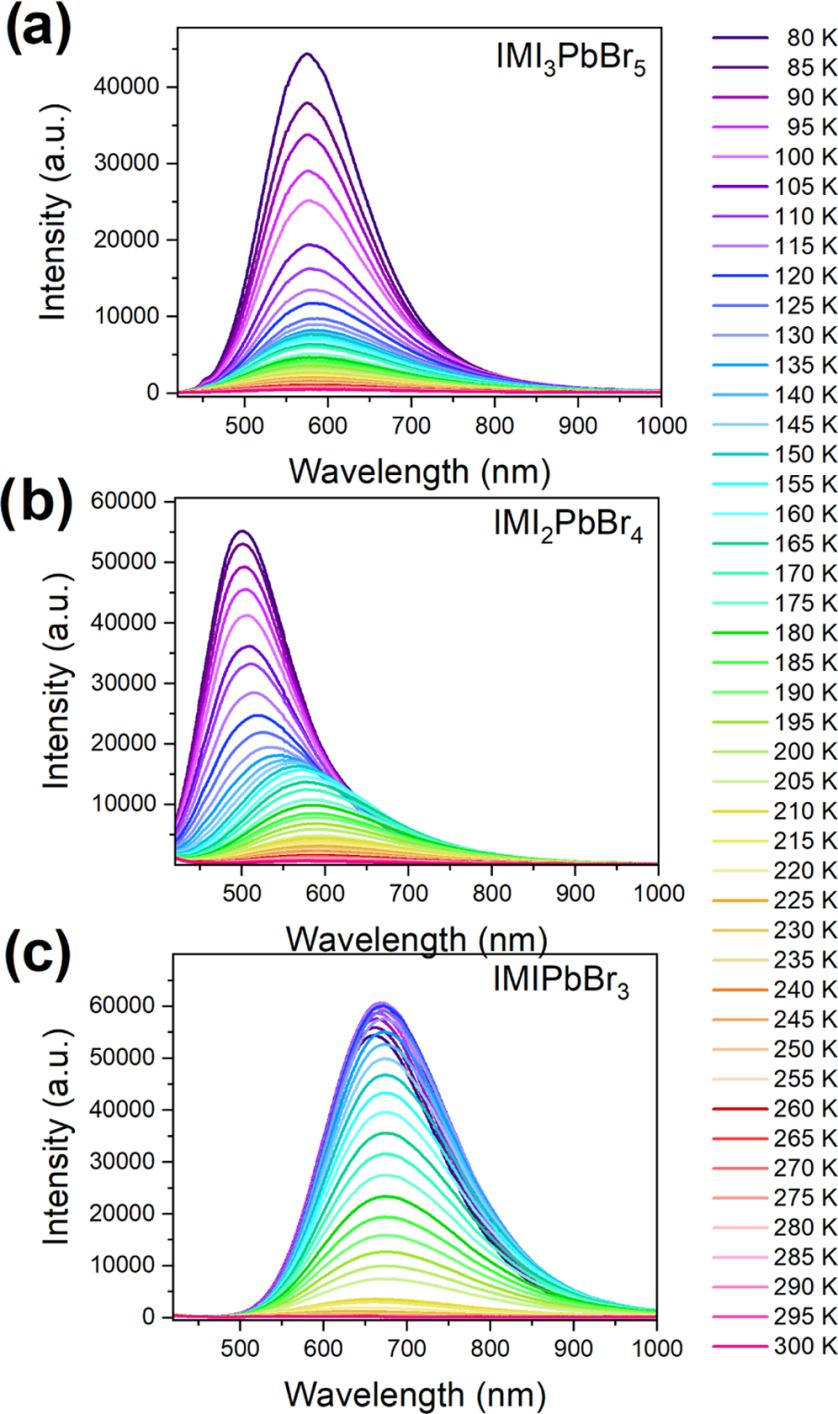
PL spectra recorded as a function of temperature for (a) IMPbBr_3_, (b) IM_2_PbBr_4_, and (c) IM_3_PbBr_5_.

The PL spectrum of IMPbBr_3_ recorded at 80 K shows an intense and very broad band (FWHM = 168 nm) centered at 668 nm (Figure 12a). The very large width and Stokes shift (271 nm, 1.26 eV) of this band suggest that it can be assigned either to the intrinsic STEs or to excitons trapped at the defects.^43,67^ It is worth adding that similar strongly Stokes-shifted and broad PL with an FWHM of about 150 nm was also reported for DMAPbBr_3_.^43^ However, in a similar manner to the excitonic absorption and band gap, the PL of DMAPbBr_3_ is also blue-shifted (to 620 nm) compared to the PL of isostructural IMPbBr_3_. Figure S16a shows that the intensity of PL of the IMPbBr_3_ sample increases on heating up to 120 K, followed by typical luminescence quenching beyond 120 K with *E*_a_ of 148 meV (Figure S17). The PL quantum yield (PLQY) of IMPbBr_3_ reaches 0.9% at RT. Anomalous enhancement of PL on heating is often observed due to the presence of phase transitions. For instance, such behavior was reported for MAPbBr_3_ and explained as resulting from reduced nonradiative recombination due to increased dielectric screening and the reduction of defects at the order–disorder phase transition.^68^ Since IMPbBr_3_ does not exhibit any phase transition near 120 K and the dielectric data do not show any increase of the dielectric permittivity near 120 K, this explanation cannot be adopted. We suppose, therefore, that the observed behavior can be explained in the same way as proposed for a few other lead bromide perovskites exhibiting anomalous temperature dependence of the PL intensity, *i.e*., this behavior can be attributed to thermally activated trapping–detrapping of excitons.^63,64,69^

IM_2_PbBr_4_ also exhibits very broad PL (FWHM = 124 nm), which can be attributed to the intrinsic STEs or excitons trapped at the defects (Figure 12b). However, the maximum of PL is blue-shifted (to 501 nm) and the Stokes shift (125 nm, 0.82 eV) is smaller as for IMPbBr_3_. The intensity of the observed PL decreases rapidly on heating with the activation energy *E*_a_ of 103 meV (Figures S16b and S18). The PL quantum yield (PLQY) of IM_2_PbBr_4_ reaches 1.9% at RT.

Broadband PL (FWHM = 140 nm) centered at 574 nm is also observed for IMI_3_PbBr_5_ (Figure 12c). This PL also decreases rapidly on heating with the activation energy *E*_a_ = 77 meV (Figures S16c and S19). The PL quantum yield (PLQY) of IMI_3_PbBr_5_ reaches 1.5% at RT.

**Figure 13:**
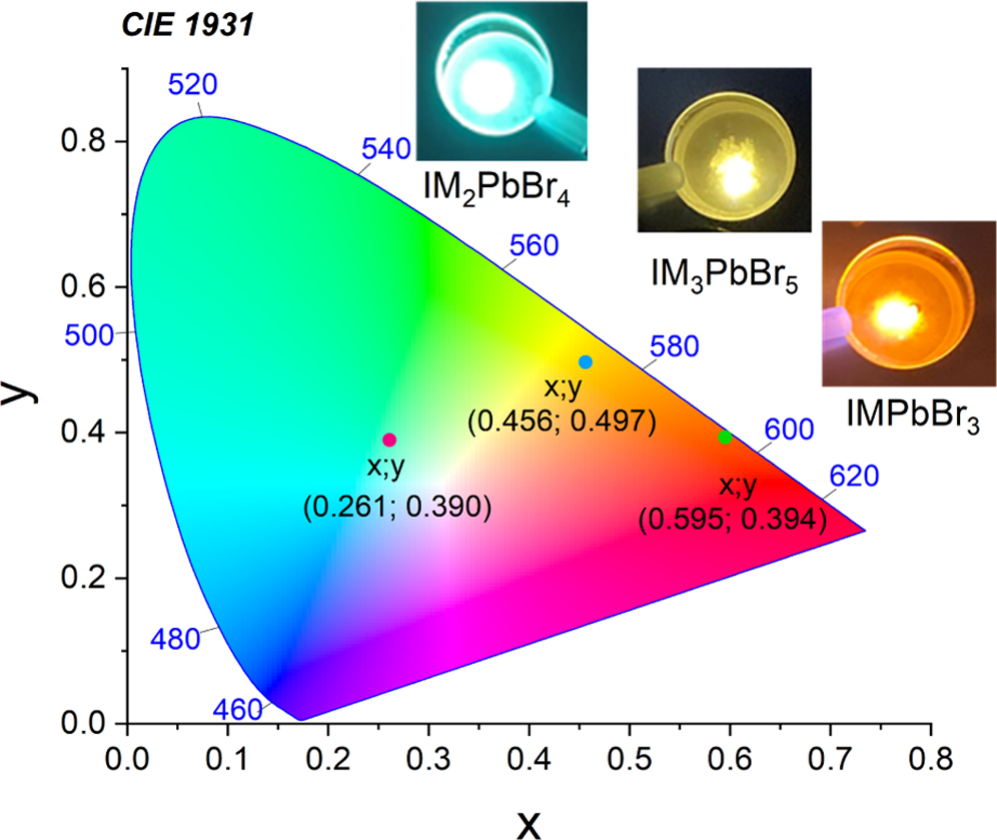
CIE coordinates of IMPbBr_3_, IM_2_PbBr_4_, and IM_3_PbBr_5_.

The PL data show that all studied compounds exhibit broadband PL. However, the maximum of PL (Stokes shift) depends strongly on the chemical composition, *i.e*., it changes from 668 nm (278 nm, 1.26 eV) for IMPbBr_3_ to 574 nm (198 nm, 1.12 eV) for IM_3_PbBr_5_ and 501 nm (125 nm, 0.82 eV) for IM_2_PbBr_4_. Table 1 shows that the bond-length distortions Δ*d* and Br–Pb–Br angles are comparable for all compounds. Octahedral angle variance (σ^2^) is the largest for IMPbBr_3_ but small and similar for IM_2_PbBr_4_ and IM_3_PbBr_5._ Thus, the difference in the distortion parameters does not explain a very large difference in the Stokes shift observed for the studied compounds. We conclude therefore that the different behavior of each composition can be attributed mainly to the different connectivity of PbBr_6_ octahedral units in the studied compounds, *i.e*., whereas in IMPbBr_3_, each PbBr_6_ octahedron is linked to four nearest neighbors (one by face-sharing and three by corner-sharing), in IM_3_PbBr_5_, all PbBr_6_ octahedra are corner-sharing, forming 1D chains, while in IM_2_PbBr_4_, the PbBr_6_ octahedra form edge-shared dimers, which then join by corners forming extending chains. The shift in the registered PL bands leads to a change in the emission color from orange for IMPbBr_3_, yellow for IM_3_PbBr_5_, and bluish-green for IM_2_PbBr_4_ (see the chromaticity CIE of the investigated compounds and the photographs of the samples’ emission in Figure 13).

## Conclusions

We have synthesized three imidazolium lead bromides: previously reported IMPbBr_3_ and two novel IM_2_PbBr_4_ and IM_3_PbBr_5_ compounds. These compounds have been studied using various experimental methods to monitor their crystal structures, mechanism of phase transitions, and dielectric and optical properties.

Single-crystal X-ray diffraction revealed that crystal structures change strongly with the chemical composition. IMPbBr_3_ crystallizes in the 4H perovskite structure with face- and corner-shared PbBr_6_ octahedra (space group *P*6_3_/*mmc*), with disordered IM^+^ cations. On cooling, it exhibits phase transitions at 240 and 200 K associated with an ordering of IM^+^ cations. Most interestingly, the LT phase is polar, *Pna*2_1_. The noncentrosymmetric nature of this phase was confirmed by its SHG activity. IM_2_PbBr_4_ adopts a 1D double-chain structure with edge-shared octahedra. At high temperatures, IM^+^ cations are disordered and the symmetry is orthorhombic (*Cmcm*). On cooling, a phase transition to the ordered *P*1̅ phase occurs at 401 K. Another isostructural and displacive-type phase transition occurs at 178 K. IM_3_PbBr_5_ crystallizes in the 1D single-chain structure with corner-shared PbBr_6_ octahedra. The crystal structure of the HT phase is orthorhombic (space group *Cmmm*), and IM^+^ cations are disordered. These cations exhibit partial ordering at 395 K, and the symmetry changes to *P*1̅. On further cooling, IM_3_PbBr_5_ transforms at 141 K to the ordered polar phase of the *P*1 symmetry, which is SHG active.

Dielectric studies revealed the presence of dielectric relaxations in all compounds. Most interestingly, IMPbBr_3_ exhibits a significant steplike dielectric anomaly at 200 K, allowing us to classify this bromide as a switchable dielectric. Furthermore, the observed change in dielectric permittivity of IM_3_PbBr_5_ near 145 K indicated that this compound is an improper ferroelectric.

Linear optical studies revealed that the excitonic bands are centered at 397 (3.12), 376 (3.30), and 378 (3.28) nm (eV) for IMPbBr_3_, IM_2_PbBr_4_, and IM_3_PbBr_5_, respectively. The band gaps are 3.24, 3.58, and 3.52 eV. All compounds exhibit broadband highly Stokes-shifted PL attributed either to the intrinsic STEx states or to excitons trapped at the defects (orange with a maximum at 668 nm for IMPbBr_3_, bluish-green with a maximum at 501 nm for IM_2_PbBr_4_, and yellow with a maximum at 574 nm for IM_3_PbBr_5_). We attributed the different behavior of each composition to the different connectivity of PbBr_6_ octahedral units in the studied compounds.

## Supporting Information Available

Crystallographic data, photos of the grown crystals, PXRD patterns, DSC traces, NLO spectra, dielectric data, Raman spectra, diffuse reflectance spectra, energy band gaps, temperature dependence of integrated PL intensities, activation energies (PDF)

## Notes

Single-crystal, powder XRD data, Raman, absorption, and emission spectra, DSC and dielectric measurements, and SHG data are available from [10.5281/zenodo.6806225] (link will be activated upon acceptance).
