# Supplementary material for: Effect of Dimensionality on Photoluminescence and Dielectric Properties of Imidazolium Lead Bromides
Source: Inorg Chem. 2022 Sep 14;61(38):15225–38. doi: 10.1021/acs.inorgchem.2c02496 (PMC9516686; doi:10.1021/acs.inorgchem.2c02496)
Supplement: Supplementary file 1 — ic2c02496_si_001.pdf [file ic2c02496_si_001.pdf]

Supporting Information

Effect of Dimensionality on Photoluminescence and Dielectric Properties of Imidazolium Lead Bromides

Szymon Smółka,,† Mirosław Mączka,*,† Dawid Drozdowski,† Dagmara Stefańska,† Anna Gągor,† Adam Sieradzki,§ Jan K. Zaręba# and Maciej Ptak†*

†Institute of Low Temperature and Structure Research, Polish Academy of Sciences, ul. Okólna 2, 50-422 Wrocław, Poland

§Department of Experimental Physics, Wrocław University of Science and Technology, Wybrzeże Wyspiańskiego 27, 50-370, Wrocław, Poland

#Advanced Materials Engineering and Modeling Group, Faculty of Chemistry, Wrocław University of Science and Technology, 50-370 Wrocław, Poland

e-mail: (SS) s.smolka@intibs.pl; (MM) m.maczka@intibs.pl

Table S1. Experimental details of $IMPbBr_3$ (M_r = 516.01).

	**Phase I, 295 K**	**Phase II, 220 K**	**Phase III, 190 K**
Crystal data			
Crystal system, space group	Hexagonal, $P6_3/mmc$	Orthorhombic, $P2_12_12_1$	Orthorhombic, $Pna2_1$
a, b, c (Å)	9.0871 (5), 9.0871 (5), 13.8501 (7)	9.0432 (8), 13.7661 (7), 31.053 (3)	14.2042 (3), 8.8719 (2), 14.9262 (2)
α, β, γ (°)	90, 90, 120	90, 90, 90	90, 90, 90
V (Å^3)	990.45 (12)	3865.8 (5)	1880.97 (6)
Z	4	16	8
μ (mm^{-1})	29.07	29.79	30.61
Crystal size (mm)	0.22 × 0.18 × 0.15		
Data collection			
T_{min}, T_{max}	0.417, 1.000	0.403, 1.000	0.426, 1.000
No. of measured, independent and observed [$I > 2\sigma(I)$] reflections	3184, 426, 357	83627, 9942, 5199	27720, 3822, 3392
R_{int}	0.043	0.175	0.044
$(\sin \theta/\lambda)_{max}$ (Å^{-1})	0.625	0.689	0.625
Refinement			
$R[F^2 > 2\sigma(F^2)]$, $wR(F^2)$, S	0.026, 0.057, 1.08	0.130, 0.376, 1.05	0.026, 0.057, 1.09
No. of reflections	426	9942	3822
No. of parameters	24	198	100
No. of restraints	0	58	1
$\Delta\rho_{max}$, $\Delta\rho_{min}$ (e Å^{-3})	0.64, -0.48	16.61, -4.28	1.01, -1.46
Flack parameter	–	0.36 (6)	0.47 (3)

Table S2. Experimental details of IM_2PbBr_4 (M_r = 665.01).

	**Phase I, 415 K**	**Phase II, 295 K**	**Phase III, 150 K**
Crystal data			
Crystal system, space group	Orthorhombic, *Cmmm*	Triclinic, *P*-1	Triclinic, *P*-1
a, *b*, *c* (Å)	9.2682 (8), 27.049 (4), 6.1301 (5)	6.0676 (2), 9.3485 (4), 13.7370 (7)	6.0086 (2), 9.4176 (3), 13.4597 (5)
α, β, γ (°)	90, 90, 90	74.34 (1), 87.03 (1), 88.61 (1)	73.80 (1), 86.52 (1), 87.16 (1)
V (Å^3)	1536.8 (3)	749.2 (1)	729.6 (1)
Z	4	2	2
μ (mm^{-1})	21.36	21.90	22.49
Crystal size (mm)	0.17 × 0.11 × 0.03		
Data collection			
T_{min}, T_{max}	0.183, 1.000	0.160, 1.000	0.172, 1.000
No. of measured, independent and observed [$I > 2\sigma(I)$] reflections	2456, 1068, 712	21352, 3060, 2652	20811, 2981, 2751
R_{int}	0.032	0.045	0.038
$(\sin \theta/\lambda)_{max}$ (Å^{-1})	0.686	0.625	0.625
Refinement			
$R[F^2 > 2\sigma(F^2)]$, $wR(F^2)$, S	0.049, 0.122, 1.05	0.034, 0.084, 1.08	0.024, 0.063, 1.07
No. of reflections	1068	3060	2981
No. of parameters	41	112	136
$\Delta\rho_{max}$, $\Delta\rho_{min}$ (e Å^{-3})	1.29, -0.93	1.50, -1.19	1.45, -1.17

Table S3. Experimental details of IM_3PbBr_5 (M_r = 814.01).

	**Phase I, 400 K**	**Phase II, 295 K**	**Phase III, 100 K**
Crystal data			
Crystal system, space group	Orthorhombic, *Cmmm*	Triclinic, *P*-1	Triclinic, *P*1
a, *b*, *c* (Å)	9.271 (2), 18.346 (4), 6.1547 (8)	6.0690 (2), 9.3749 (5), 9.7338 (6)	5.9724 (3), 9.3996 (4), 9.4745 (5)
α, β, γ (°)	90, 90, 90	66.98 (1), 86.87 (1), 88.62 (1)	67.42 (1), 85.47 (1), 86.93 (1)
V (Å^3)	1046.8 (3)	508.97 (5)	489.4 (1)
Z	2	1	1
μ (mm^{-1})	17.60	18.10	18.82
Crystal size (mm)	0.13 × 0.08 × 0.03	0.13 × 0.08 × 0.03	0.13 × 0.08 × 0.03
Data collection			
T_{min}, T_{max}	0.123, 1.000	0.450, 1.000	0.366, 1.000
No. of measured, independent and observed [$I > 2\sigma(I)$] reflections	1591, 640, 487	7248, 2080, 1876	8580, 4440, 4256
R_{int}	0.046	0.036	0.025
$(\sin \theta/\lambda)_{max}$ (Å^{-1})	0.624	0.625	0.687
Refinement			
$R[F^2 > 2\sigma(F^2)]$, $wR(F^2)$, S	0.051, 0.128, 1.03	0.030, 0.066, 1.04	0.026, 0.050, 1.05
No. of reflections	640	2080	4440
No. of parameters	29	89	95
No. of restraints	0	0	3
$\Delta\rho_{max}$, $\Delta\rho_{min}$ (e Å^{-3})	1.12, -1.62	0.80, -1.15	0.93, -1.02
Flack parameter	–	–	0.50 (1)

Table S4. Selected geometric parameters of $IMPbBr_3$ (Å, º).

**Phase I, 295 K**			
Pb1—Br1	3.0223 (10)	Pb1—Br2^{ii}	3.0083 (3)
Pb1—Br1^{i}	3.0223 (10)	Pb1—Br2	3.0083 (3)
Pb1—Br1^{ii}	3.0223 (10)	Pb1—Br2^{i}	3.0083 (3)
Br1^{ii}—Pb1—Br1^{i}	81.36 (3)	Br2—Pb1—Br1^{i}	168.13 (2)
Br1—Pb1—Br1^{ii}	81.36 (3)	Br2—Pb1—Br1	89.664 (10)
Br1—Pb1—Br1^{i}	81.36 (3)	Br2^{i}—Pb1—Br1	89.664 (10)
Br2^{ii}—Pb1—Br1^{i}	89.665 (10)	Br2—Pb1—Br2^{ii}	98.080 (11)
Br2^{i}—Pb1—Br1^{i}	89.664 (10)	Br2^{i}—Pb1—Br2^{ii}	98.080 (11)
Br2—Pb1—Br1^{ii}	89.664 (10)	Br2^{i}—Pb1—Br2	98.080 (11)
Br2^{ii}—Pb1—Br1	168.13 (2)	Pb1^{iii}—Br1—Pb1	82.36 (3)
Br2^{i}—Pb1—Br1^{ii}	168.13 (2)	Pb1^{iv}—Br2—Pb1	180.0
Br2^{ii}—Pb1—Br1^{ii}	89.665 (10)		
**Phase II, 220 K**			
Pb01—Br05	3.015 (6)	Pb03—Br08	3.002 (6)
Pb01—Br06	3.024 (6)	Pb03—Br09	2.990 (6)
Pb01—Br07^{v}	3.014 (6)	Pb03—Br0A	3.017 (6)
Pb01—Br0B	2.991 (8)	Pb03—Br0C^{viii}	2.955 (7)
Pb01—Br0E	2.964 (7)	Pb03—Br0C	3.060 (7)
Pb01—Br0F	2.992 (7)	Pb03—Br0D^{vi}	3.043 (6)
Pb02—Br05^{vi}	3.045 (6)	Pb04—Br08	3.013 (7)
Pb02—Br06^{vi}	2.997 (6)	Pb04—Br09	3.023 (6)
Pb02—Br07	3.003 (6)	Pb04—Br0A	3.063 (7)
Pb02—Br0B^{vii}	3.013 (8)	Pb04—Br0F	2.956 (7)
Pb02—Br0D	3.000 (6)	Pb04—Br0G^{ix}	2.987 (10)
Pb02—Br0E	3.012 (7)	Pb04—Br0G	2.931 (9)
Br05—Pb01—Br06	81.85 (16)	Br09—Pb03—Br0D^{vi}	168.2 (2)
Br07^{v}—Pb01—Br05	81.96 (15)	Br0A—Pb03—Br0C	89.0 (2)
Br07^{v}—Pb01—Br06	82.00 (16)	Br0A—Pb03—Br0D^{vi}	90.0 (2)
Br0B—Pb01—Br05	88.1 (2)	Br0C^{viii}—Pb03—Br08	90.2 (2)
Br0B—Pb01—Br06	89.9 (2)	Br0C^{viii}—Pb03—Br09	89.3 (2)
Br0B—Pb01—Br07^{v}	167.9 (2)	Br0C^{viii}—Pb03—Br0A	168.9 (2)
Br0B—Pb01—Br0F	91.8 (3)	Br0C^{viii}—Pb03—Br0C	97.65 (6)
Br0E—Pb01—Br05	170.1 (2)	Br0C^{viii}—Pb03—Br0D^{vi}	97.2 (3)
Br0E—Pb01—Br06	90.5 (2)	Br0D^{vi}—Pb03—Br0C	102.0 (3)

Br0E—Pb01—Br07^{v}	90.7 (2)	Br08—Pb04—Br09	81.98 (17)
Br0E—Pb01—Br0B	98.3 (2)	Br08—Pb04—Br0A	80.78 (16)
Br0E—Pb01—Br0F	100.1 (3)	Br09—Pb04—Br0A	80.90 (16)
Br0F—Pb01—Br05	87.3 (2)	Br0F—Pb04—Br08	92.1 (2)
Br0F—Pb01—Br06	168.9 (2)	Br0F—Pb04—Br09	167.3 (2)
Br0F—Pb01—Br07^{v}	94.5 (2)	Br0F—Pb04—Br0A	87.1 (2)
Br06^{vi}—Pb02—Br05^{vi}	81.79 (15)	Br0F—Pb04—Br0G^{ix}	97.9 (3)
Br06^{vi}—Pb02—Br07	82.63 (16)	Br0G—Pb04—Br08	168.2 (3)
Br06^{vi}—Pb02—Br0B^{vii}	90.9 (2)	Br0G^{ix}—Pb04—Br08	90.2 (3)
Br06^{vi}—Pb02—Br0D	166.6 (2)	Br0G—Pb04—Br09	90.9 (3)
Br06^{vi}—Pb02—Br0E	88.8 (2)	Br0G^{ix}—Pb04—Br09	93.4 (3)
Br07—Pb02—Br05^{vi}	81.64 (15)	Br0G—Pb04—Br0A	88.8 (3)
Br07—Pb02—Br0B^{vii}	169.1 (2)	Br0G^{ix}—Pb04—Br0A	169.9 (3)
Br07—Pb02—Br0E	90.5 (2)	Br0G—Pb04—Br0F	92.9 (3)
Br0B^{vii}—Pb02—Br05^{vi}	88.8 (2)	Br0G—Pb04—Br0G^{ix}	99.69 (7)
Br0D—Pb02—Br05^{vi}	88.2 (2)	Pb01—Br05—Pb02^{v}	81.09 (13)
Br0D—Pb02—Br07	87.1 (2)	Pb02^{v}—Br06—Pb01	81.72 (15)
Br0D—Pb02—Br0B^{vii}	97.8 (3)	Pb02—Br07—Pb01^{vi}	81.77 (14)
Br0D—Pb02—Br0E	100.0 (3)	Pb03—Br08—Pb04	82.22 (15)
Br0E—Pb02—Br05^{vi}	168.4 (2)	Pb03—Br09—Pb04	82.25 (16)
Br0E—Pb02—Br0B^{vii}	98.1 (2)	Pb03—Br0A—Pb04	81.16 (16)
Br08—Pb03—Br0A	81.70 (17)	Pb01—Br0B—Pb02^{x}	172.3 (4)
Br08—Pb03—Br0C	166.8 (2)	Pb03^{xi}—Br0C—Pb03	174.8 (4)
Br08—Pb03—Br0D^{vi}	87.4 (2)	Pb02—Br0D—Pb03^{v}	175.9 (3)
Br09—Pb03—Br08	82.72 (17)	Pb01—Br0E—Pb02	174.4 (4)
Br09—Pb03—Br0A	82.19 (18)	Pb04—Br0F—Pb01	176.6 (4)
Br09—Pb03—Br0C	86.8 (2)	Pb04—Br0G—Pb04^{xii}	176.4 (5)
**Phase III, 190 K**			
Pb1—Br1	3.051 (5)	Pb2—Br1^{xv}	3.097 (4)
Pb1—Br2	2.969 (3)	Pb2—Br2	2.955 (3)
Pb1—Br6^{xiii}	3.055 (6)	Pb2—Br6	2.979 (6)
Pb1—Br3	3.010 (4)	Pb2—Br3	2.980 (4)
Pb1—Br4	3.028 (4)	Pb2—Br4	3.020 (4)
Pb1—Br5^{xiv}	3.094 (4)	Pb2—Br5	3.051 (4)
Br1—Pb1—Br6^{xiii}	88.06 (13)	Br2—Pb2—Br4	82.67 (9)
Br1—Pb1—Br5^{xiv}	92.71 (17)	Br2—Pb2—Br5	93.13 (10)
Br2—Pb1—Br1	93.52 (10)	Br6—Pb2—Br1^{xv}	86.24 (14)

Br2—Pb1—Br6^{xiii}	90.92 (13)	Br6—Pb2—Br3	174.01 (14)
Br2—Pb1—Br3	82.33 (9)	Br6—Pb2—Br4	98.56 (14)
Br2—Pb1—Br4	82.30 (9)	Br6—Pb2—Br5	88.59 (14)
Br2—Pb1—Br5^{xiv}	173.14 (10)	Br3—Pb2—Br1^{xv}	99.74 (10)
Br6^{xiii}—Pb1—Br5^{xiv}	86.43 (14)	Br3—Pb2—Br4	81.77 (8)
Br3—Pb1—Br1	91.33 (9)	Br3—Pb2—Br5	90.69 (9)
Br3—Pb1—Br6^{xiii}	173.18 (13)	Br4—Pb2—Br1^{xv}	91.24 (11)
Br3—Pb1—Br4	81.14 (9)	Br4—Pb2—Br5	171.74 (11)
Br3—Pb1—Br5^{xiv}	100.39 (9)	Br5—Pb2—Br1^{xv}	93.36 (17)
Br4—Pb1—Br1	171.80 (11)	Pb1—Br1—Pb2^{xvi}	169.41 (18)
Br4—Pb1—Br6^{xiii}	99.02 (13)	Pb2—Br2—Pb1	82.24 (2)
Br4—Pb1—Br5^{xiv}	91.86 (11)	Pb2—Br6—Pb1^{xvii}	179.4 (2)
Br2—Pb2—Br1^{xv}	172.88 (10)	Pb2—Br3—Pb1	81.14 (2)
Br2—Pb2—Br6	91.01 (13)	Pb2—Br4—Pb1	80.21 (2)
Br2—Pb2—Br3	83.09 (9)	Pb2—Br5—Pb1^{xviii}	168.93 (17)

Symmetry code(s): (i) -*x*+*y*, -*x*+1, *z*; (ii) -*y*+1, *x*-*y*+1, *z*; (iii) *x*, *y*, -*z*+1/2; (iv) -*x*, -*y*+1, -*z*+1; (v) -*x*+1, *y*-1/2, -*z*+1/2; (vi) -*x*+1, *y*+1/2, -*z*+1/2; (vii) *x*-1, *y*, *z*; (viii) *x*+1/2, -*y*+3/2, -*z*+1; (ix) *x*+1/2, -*y*+1/2, -*z*+1; (x) *x*+1, *y*, *z*; (xi) *x*-1/2, -*y*+3/2, -*z*+1; (xii) *x*-1/2, -*y*+1/2, -*z*+1; (xiii) -*x*, -*y*, *z*-1/2; (xiv) -*x*+1/2, *y*-1/2, *z*-1/2; (xv) -*x*+1/2, *y*-1/2, *z*+1/2; (xvi) -*x*+1/2, *y*+1/2, *z*-1/2; (xvii) -*x*, -*y*, *z*+1/2; (xviii) -*x*+1/2, *y*+1/2, *z*+1/2.

Table S5. Selected geometric parameters of IM_2PbBr_4 (Å, º).

Phase I, 415 K		Phase II, 295 K		Phase III, 150 K	
Pb1—Br4^{i}	3.0761 (14)	Pb1—Br4^{i}	3.0897 (9)	Pb1—Br4^{i}	3.0972 (6)
Pb1—Br4	3.0761 (14)	Pb1—Br4	3.0484 (9)	Pb1—Br4	2.9872 (6)
Pb1—Br1	3.0651 (3)	Pb1—Br1^{v}	3.0495 (8)	Pb1—Br1^{v}	3.0732 (6)
Pb1—Br1^{ii}	3.0651 (3)	Pb1—Br1	3.0187 (8)	Pb1—Br1	2.9492 (6)
Pb1—Br2	2.9531 (17)	Pb1—Br2	2.9485 (9)	Pb1—Br2	2.9321 (6)
Pb1—Br2^{iii}	2.9530 (17)	Pb1—Br3	2.9937 (9)	Pb1—Br3	3.0576 (6)
Br4—Pb1—Br4^{i}	85.19 (5)	Br4—Pb1—Br4^{i}	86.44 (2)	Br4—Pb1—Br4^{i}	88.041 (16)
Br1—Pb1—Br4	90.33 (4)	Br4—Pb1—Br1^{v}	88.24 (2)	Br4—Pb1—Br1^{v}	85.715 (16)
Br1—Pb1—Br4^{i}	90.33 (4)	Br1—Pb1—Br4^{i}	89.79 (2)	Br4—Pb1—Br3	177.891 (17)
Br1^{ii}—Pb1—Br4^{i}	90.33 (4)	Br1—Pb1—Br4	91.61 (3)	Br1^{v}—Pb1—Br4^{i}	92.642 (16)
Br1^{ii}—Pb1—Br4	90.33 (4)	Br1^{v}—Pb1—Br4^{i}	88.55 (2)	Br1—Pb1—Br4	87.435 (17)
Br1—Pb1—Br1^{ii}	179.11 (11)	Br1—Pb1—Br1^{v}	178.35 (4)	Br1—Pb1—Br4^{i}	90.812 (17)
Br2—Pb1—Br4	176.51 (5)	Br2—Pb1—Br4^{i}	177.00 (3)	Br1—Pb1—Br1^{v}	172.22 (2)
Br2—Pb1—Br4^{i}	91.32 (5)	Br2—Pb1—Br4	90.60 (3)	Br1—Pb1—Br3	91.699 (17)
Br2^{iii}—Pb1—Br4	91.32 (5)	Br2—Pb1—Br1^{v}	91.80 (3)	Br2—Pb1—Br4^{i}	177.706 (17)
Br2^{iii}—Pb1—Br4^{i}	176.51 (5)	Br2—Pb1—Br1	89.85 (3)	Br2—Pb1—Br4	90.955 (17)
Br2^{iii}—Pb1—Br1	89.69 (4)	Br2—Pb1—Br3	90.80 (3)	Br2—Pb1—Br1^{v}	89.339 (17)
Br2—Pb1—Br1	89.69 (4)	Br3—Pb1—Br4^{i}	92.17 (2)	Br2—Pb1—Br1	87.081 (17)
Br2—Pb1—Br1^{ii}	89.69 (4)	Br3—Pb1—Br4	178.59 (3)	Br2—Pb1—Br3	90.921 (18)
Br2^{iii}—Pb1—Br1^{ii}	89.69 (4)	Br3—Pb1—Br1	88.59 (3)	Br3—Pb1—Br4^{i}	90.050 (16)
Br2^{iii}—Pb1—Br2	92.17 (8)	Br3—Pb1—Br1^{v}	91.52 (3)	Br3—Pb1—Br1^{v}	95.270 (17)
Pb1^{i}—Br4—Pb1	94.81 (5)	Pb1—Br4—Pb1^{i}	93.56 (2)	Pb1—Br4—Pb1^{i}	91.961 (16)
Pb1—Br1—Pb1^{iv}	179.11 (11)	Pb1—Br1—Pb1^{vi}	178.35 (4)	Pb1—Br1—Pb1^{vi}	172.21 (2)

Symmetry code(s): (i) $-x$+1, $-y$+1, $-z$+1; (ii) x, y, z+1; (iii) $-x$+1, y, $-z$+1; (iv) x, y, z-1; (v) x+1, y, z; (vi) x-1, y, z.

Table S6. Selected geometric parameters of IM_3PbBr_5 (Å, º).

**Phase I, 400 K**		**Phase II, 295 K**		**Phase III, 100 K**	
Pb1—Br1	3.0774 (4)	Pb1—Br3^{vi}	3.0324 (7)	Pb1—Br2	3.0153 (11)
Pb1—Br1^{i}	3.0774 (4)	Pb1—Br3	3.0324 (7)	Pb1—Br4	3.0058 (12)
Pb1—Br2^{ii}	3.0107 (19)	Pb1—Br2	3.0097 (7)	Pb1—Br1	3.0305 (13)
Pb1—Br2^{iii}	3.0107 (19)	Pb1—Br2^{vi}	3.0097 (7)	Pb1—Br1^{vii}	2.9641 (13)
Pb1—Br2	3.0107 (19)	Pb1—Br1^{vii}	3.0345 (1)	Pb1—Br3	3.0608 (12)
Pb1—Br2^{iv}	3.0107 (19)	Pb1—Br1	3.0345 (1)	Pb1—Br5	2.9688 (11)
Br1^{i}—Pb1—Br1	180.0	Br3—Pb1—Br3^{vi}	180.0	Br2—Pb1—Br1	86.97 (3)
Br2^{ii}—Pb1—Br1	90.0	Br3—Pb1—Br1^{vii}	88.500 (13)	Br2—Pb1—Br3	88.48 (3)
Br2^{iv}—Pb1—Br1^{i}	90.0	Br3^{vi}—Pb1—Br1	88.500 (13)	Br4—Pb1—Br2	91.92 (3)
Br2—Pb1—Br1	90.0	Br3^{vi}—Pb1—Br1^{vii}	91.500 (13)	Br4—Pb1—Br1	86.88 (4)
Br2^{iv}—Pb1—Br1	90.0	Br3—Pb1—Br1	91.500 (13)	Br4—Pb1—Br3	177.94 (5)
Br2—Pb1—Br1^{i}	90.0	Br2^{vi}—Pb1—Br3^{vi}	88.35 (2)	Br1^{vii}—Pb1—Br2	85.06 (3)
Br2^{iii}—Pb1—Br1	90.0	Br2—Pb1—Br3^{vi}	91.65 (2)	Br1^{vii}—Pb1—Br4	87.59 (3)
Br2^{iii}—Pb1—Br1^{i}	90.0	Br2^{vi}—Pb1—Br3	91.65 (2)	Br1^{vii}—Pb1—Br1	170.14 (3)
Br2^{ii}—Pb1—Br1^{i}	90.0	Br2—Pb1—Br3	88.35 (2)	Br1—Pb1—Br3	95.15 (3)
Br2—Pb1—Br2^{iv}	88.94 (8)	Br2—Pb1—Br2^{vi}	180.0	Br1^{vii}—Pb1—Br3	90.43 (3)
Br2^{ii}—Pb1—Br2^{iv}	180.0	Br2—Pb1—Br1^{vii}	89.767 (13)	Br1^{vii}—Pb1—Br5	92.89 (3)
Br2^{iii}—Pb1—Br2^{ii}	88.94 (8)	Br2—Pb1—Br1	90.233 (13)	Br5—Pb1—Br2	177.56 (4)
Br2—Pb1—Br2^{iii}	180.0	Br2^{vi}—Pb1—Br1^{vii}	90.234 (13)	Br5—Pb1—Br4	89.31 (3)
Br2^{iii}—Pb1—Br2^{iv}	91.06 (8)	Br2^{vi}—Pb1—Br1	89.766 (13)	Br5—Pb1—Br1	95.20 (3)
Br2—Pb1—Br2^{ii}	91.06 (8)	Br1^{vii}—Pb1—Br1	180.0	Br5—Pb1—Br3	90.22 (3)
Pb1^{v}—Br1—Pb1	180.0	Pb1^{viii}—Br1—Pb1	180.0	Pb1^{viii}—Br1—Pb1	170.14 (3)

Symmetry code(s): (i) *x*, *y*, *z*+1; (ii) *x*, -*y*+1, *z*; (iii) -*x*+1, -*y*+1, -*z*+1; (iv) -*x*+1, *y*, -*z*+1; (v) *x*, *y*, *z*-1; (vi) -*x*+1, -*y*+1, -*z*; (vii) *x*+1, *y*, *z*; (viii) *x*-1, *y*, *z*.

Table S7. Selected hydrogen-bond parameters of $IMPbBr_3$ (phase **III**).

D—H···*A*	*D*—H (Å)	H···*A* (Å)	*D*···*A* (Å)	*D*—H···*A* (°)
N3—H3···Br5	0.86	2.91	3.535 (9)	131.1
N4—H4···Br1	0.86	2.61	3.437 (9)	162.0
N1—H1···Br4^{i}	0.86	2.83	3.662 (9)	163.2
N2—H2···Br5^{ii}	0.86	2.74	3.461 (10)	142.8

Symmetry code(s): (i) -*x*+1/2, *y*+1/2, *z*-1/2; (ii) -*x*+1, -*y*, *z*-1/2.

Table S8. Selected hydrogen-bond parameters of IM_2PbBr_4.

D—H···*A*	*D*—H (Å)	H···*A* (Å)	*D*···*A* (Å)	*D*—H···*A* (°)
**Phase II, 295 K**				
N5—H5···Br3^{i}	0.86	2.86	3.478 (11)	130.0
N5—H5···Br3^{ii}	0.86	2.93	3.544 (11)	129.5
N10—H10···Br3^{iii}	0.86	2.72	3.561 (11)	166.3
N2—H2···Br2	0.86	2.58	3.397 (9)	158.4
**Phase III, 150 K**				
N5—H5···Br3^{i}	0.86	2.83	3.429 (6)	127.9
N5—H5···Br3^{ii}	0.86	2.82	3.452 (5)	131.8
N10—H10···Br3^{iii}	0.86	2.54	3.365 (5)	160.2
N2—H2···Br2	0.86	2.47	3.312 (5)	165.4
N7—H7···Br4^{iv}	0.86	2.92	3.484 (6)	125.1
N7—H7···Br1^{iv}	0.86	2.75	3.472 (5)	141.9

Symmetry code(s): (i) *x*-1, *y*, *z*; (ii) -*x*, -*y*+1, -*z*; (iii) -*x*+2, -*y*+1, -*z*+1; (iv) -*x*+1, -*y*+2, -*z*+1.

Table S9. Selected hydrogen-bond parameters of IM_3PbBr_5.

D—H···*A*	*D*—H (Å)	H···*A* (Å)	*D*···*A* (Å)	*D*—H···*A* (°)
**Phase II, 295 K**				
N8—H8···Br3^{i}	0.86	2.82	3.464 (6)	133.2
N10—H10···Br2	0.86	2.56	3.377 (7)	158.3
**Phase III, 150 K**				
N8—H8···Br3^{i}	0.86	2.87	3.435 (7)	124.6
N10—H10···Br2	0.86	2.46	3.285 (7)	161.8
N13—H13···Br3^{ii}	0.86	2.86	3.465 (7)	129.0

Symmetry code(s): (i) *x*+1, *y*, *z*; (ii) *x*-1, *y*, *z*.

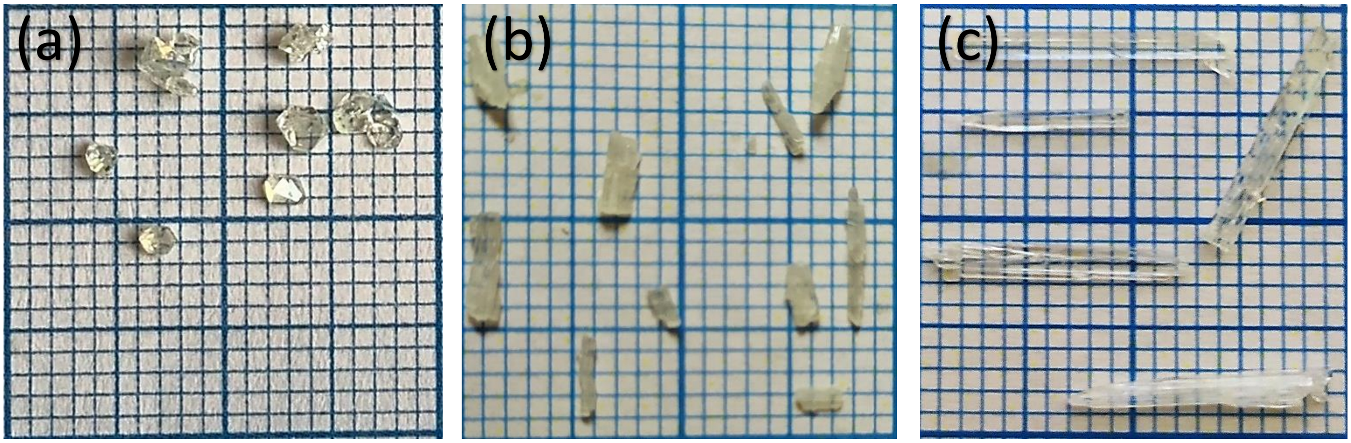

Figure S1. Photos of the grown (a) $IMPbBr_3$, (b) IM_2PbBr_4 and (c) IM_3PbBr_5 crystals.

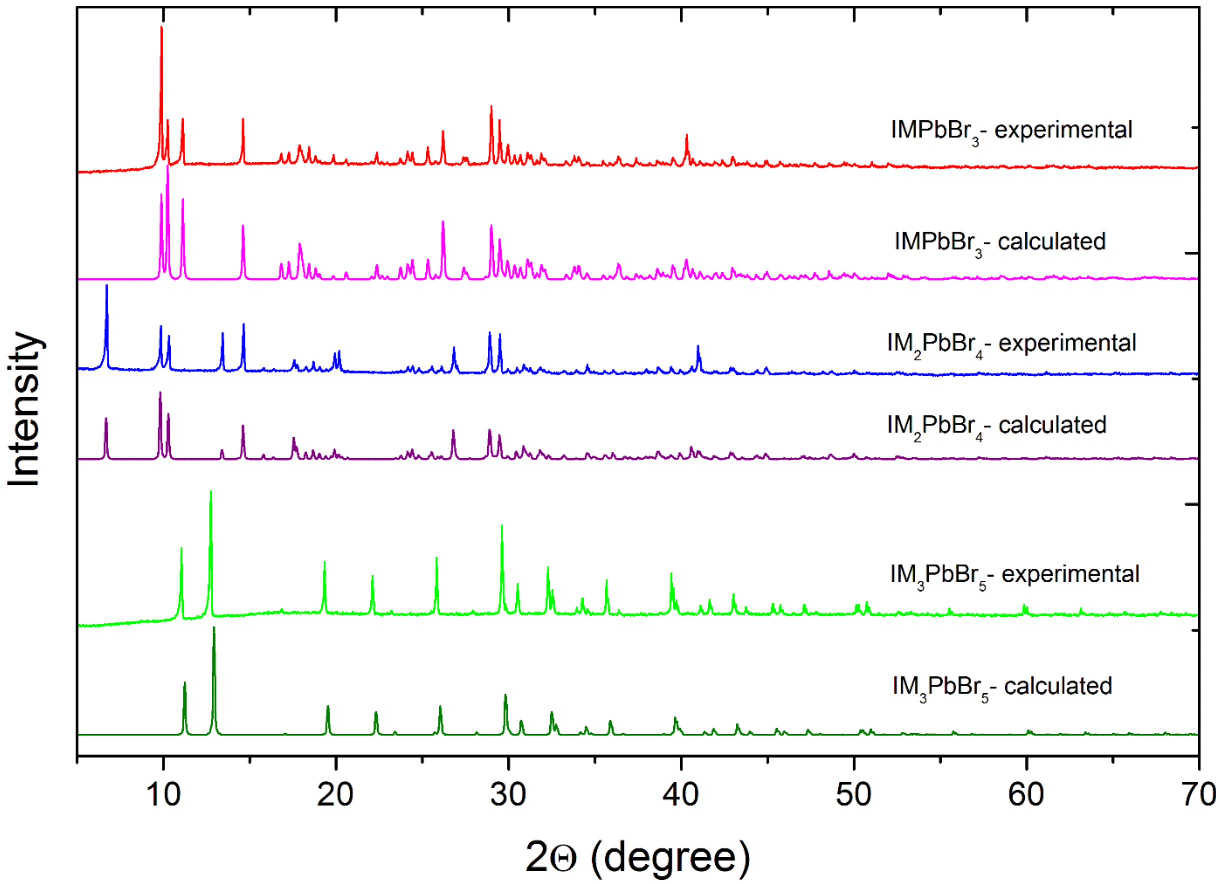

Figure S2. Powder XRD patterns for $IMPbBr_3$, IM_2PbBr_4 and IM_3PbBr_5 together with the calculated ones based on the RT single crystal structures.

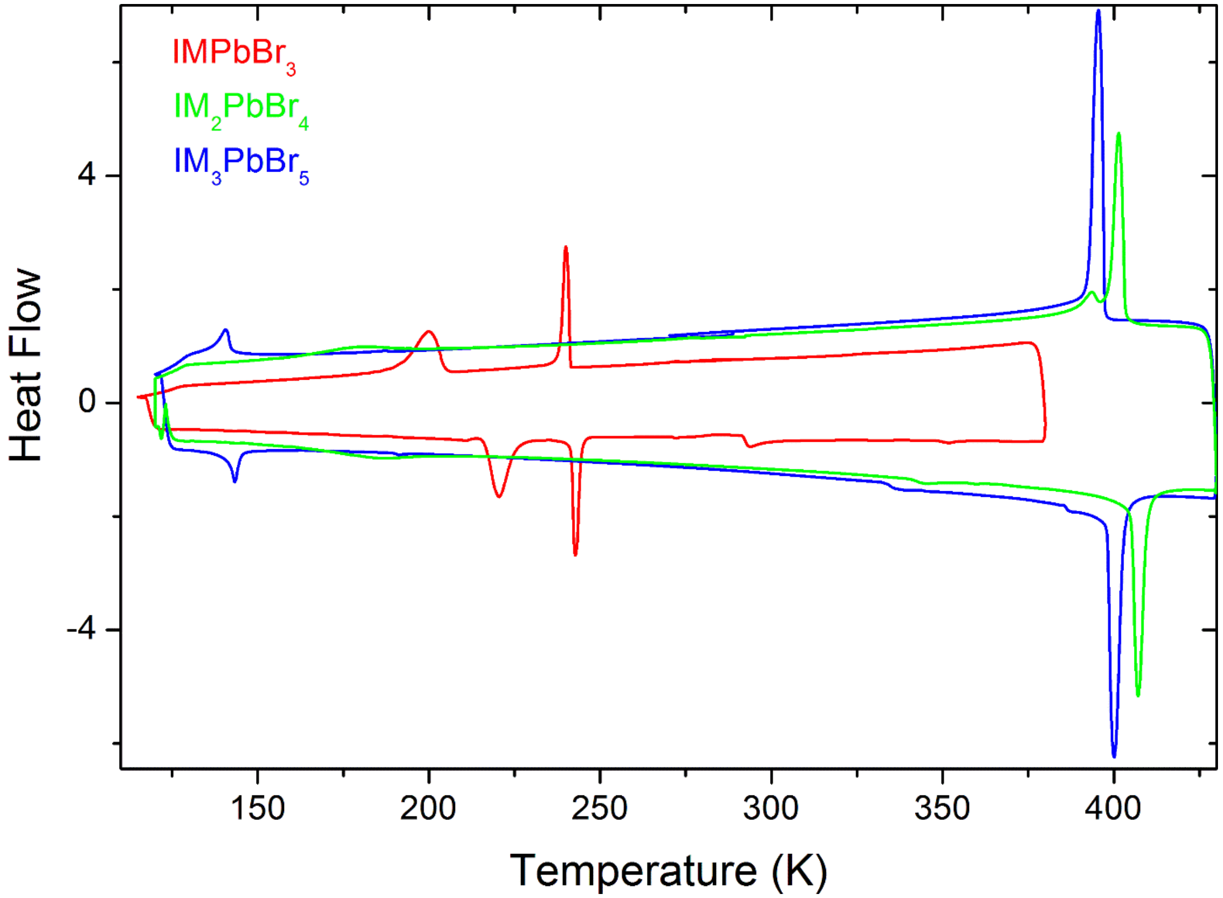

Figure S3. DSC traces for $IMPbBr_3$ (red line), IM_2PbBr_4 (green line) and IM_3PbBr_5 (blue line) in heating and cooling modes and the temperature range of 110-430 K.

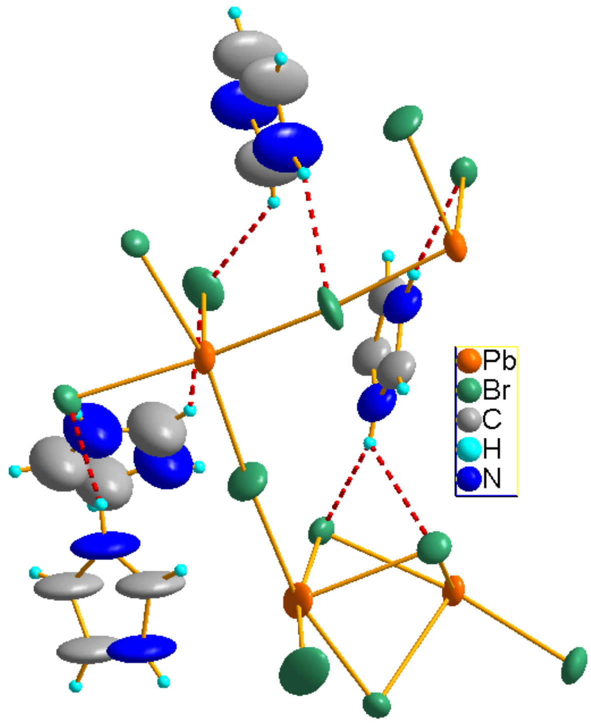

Figure S4. The asymmetric unit in the IMPbBr$_3$, phase **II**, orthorhombic $P2_12_12_1$. The dashed red lines denote hydrogen bonds.

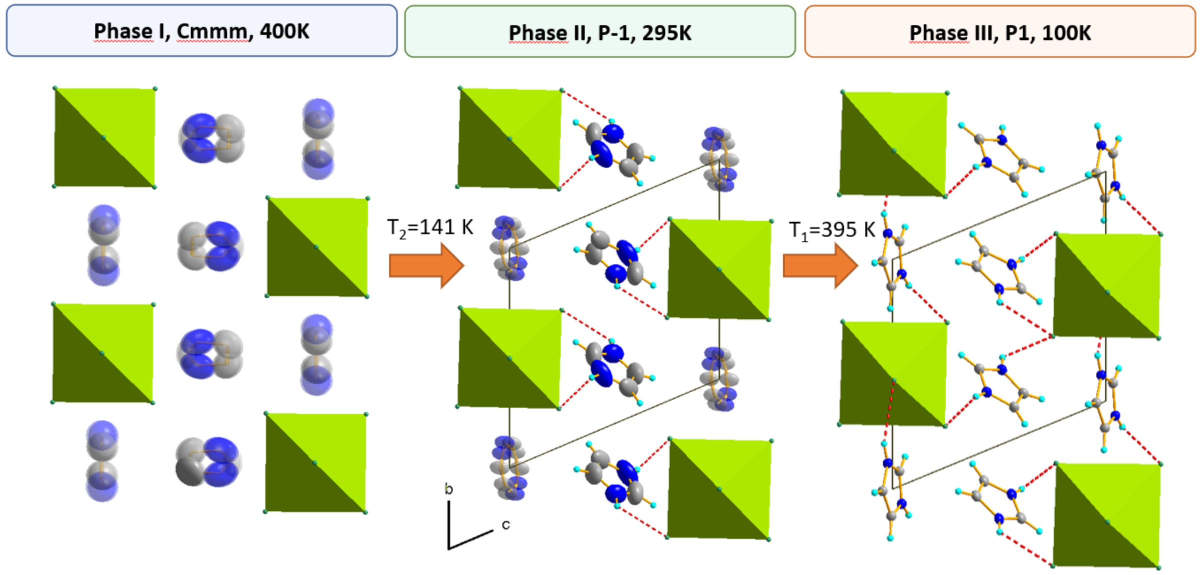

Figure S5. Phase transitions in IM$_3$PbBr$_5$**.**

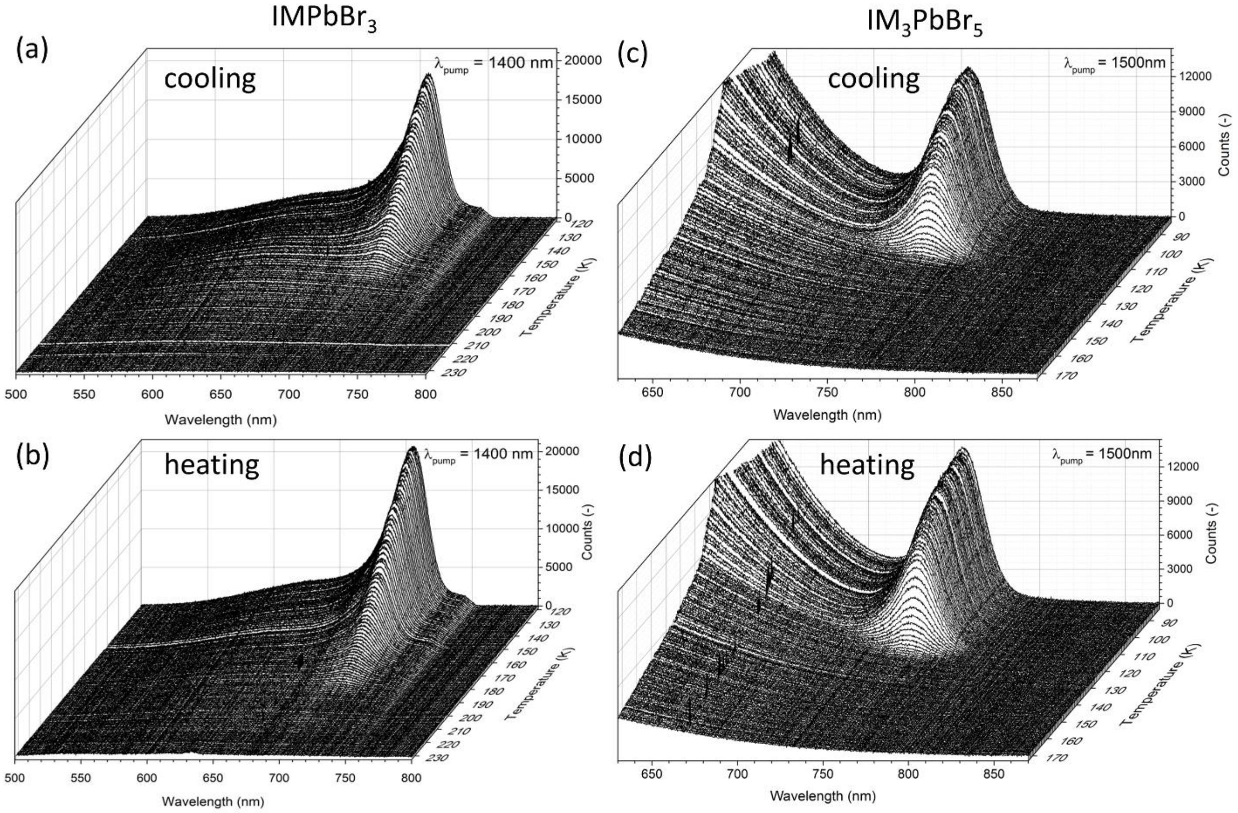

Figure S6. Experimental spectra of NLO responses of (a,b) $IMPbBr_3$ and (c,d) IM_3PbBr_5 for (a,c) cooling run and (b,d) heating run plotted as a function of temperature.

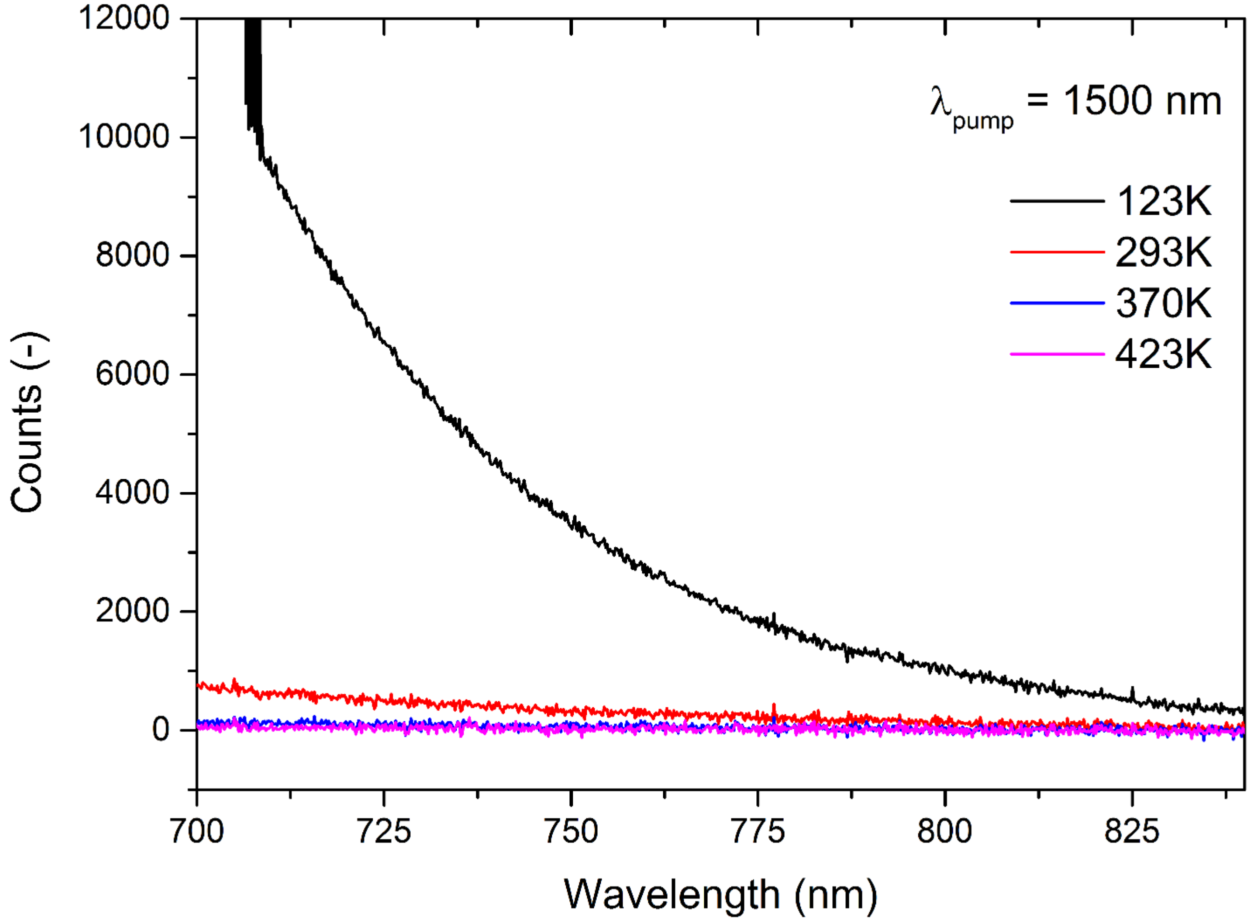

Figure S7. Experimental spectra of NLO responses of IM_2PbBr_4 for selected temperatures. Note that only for 123K and 293K tails of MPEL can be observed, while no SHG signal is present at 750 nm at any temperature.

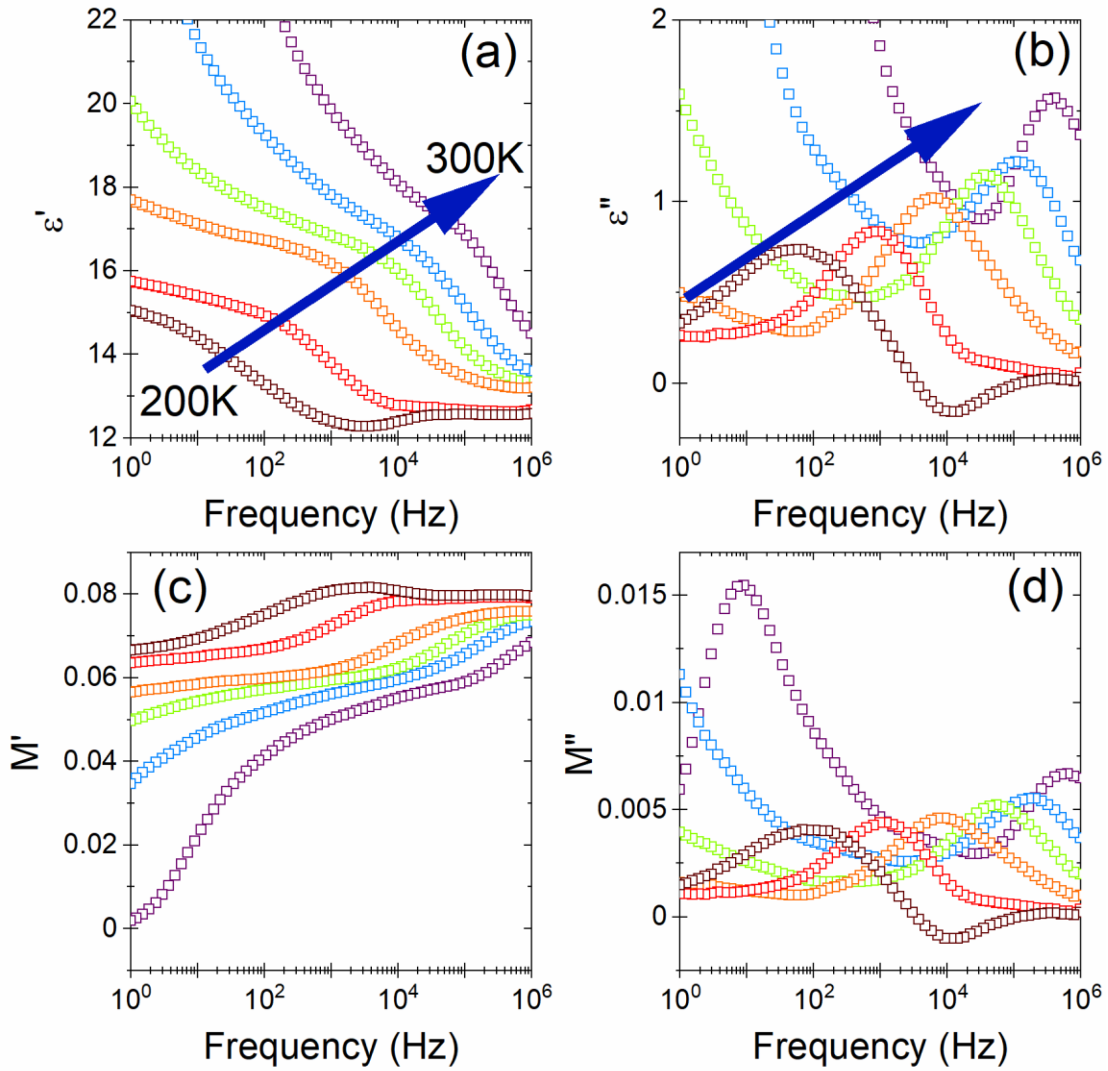

Figure S8. The frequency dependence of dielectric permittivity (a), dielectric loss (b), real (c) and imaginary (d) part of electric modulus spectra of $IMPbBr_3$.

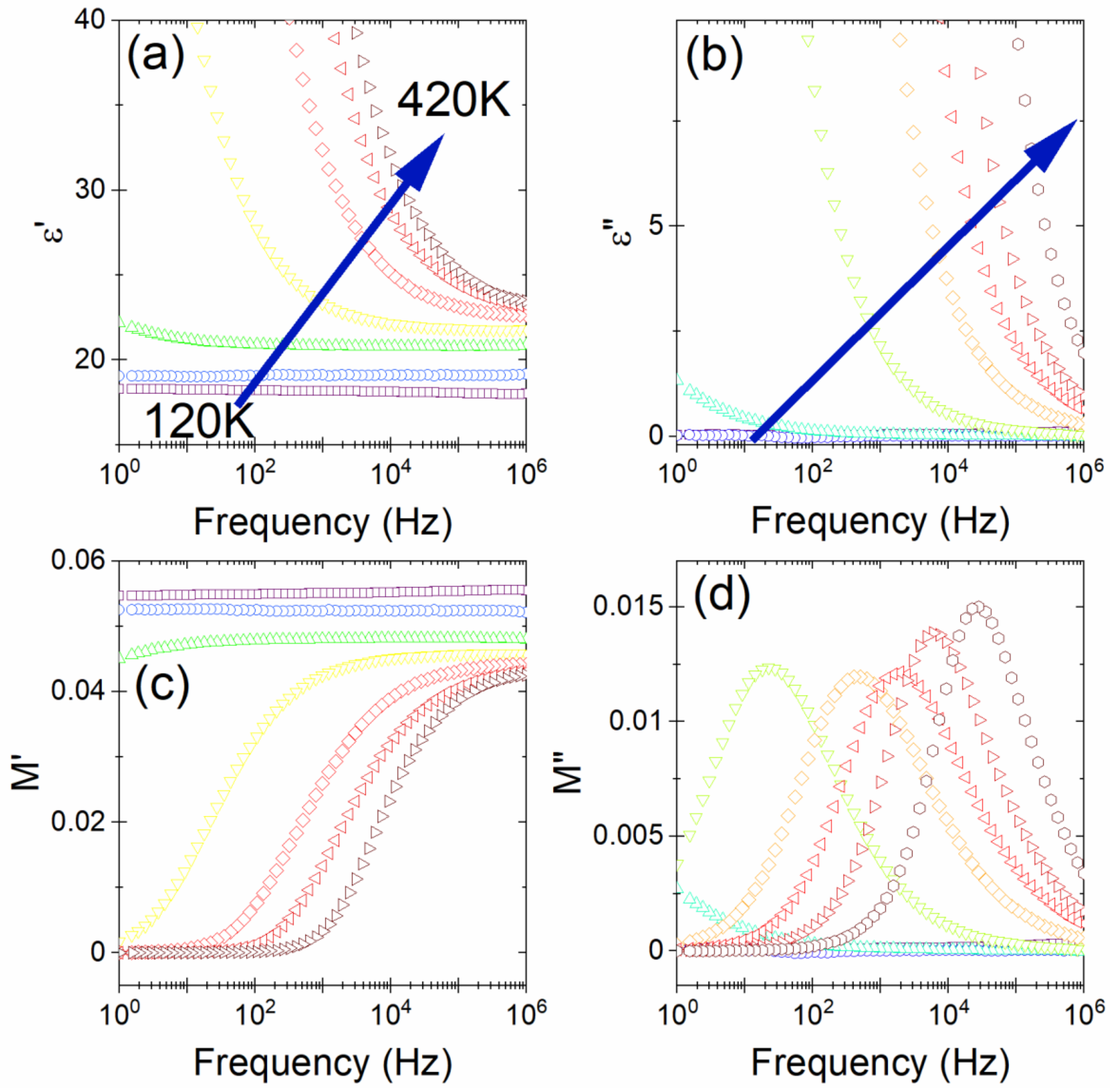

Figure S9. The frequency dependence of dielectric permittivity (a), dielectric loss (b), real (c) and imaginary (d) part of electric modulus spectra of IM_2PbBr_4.

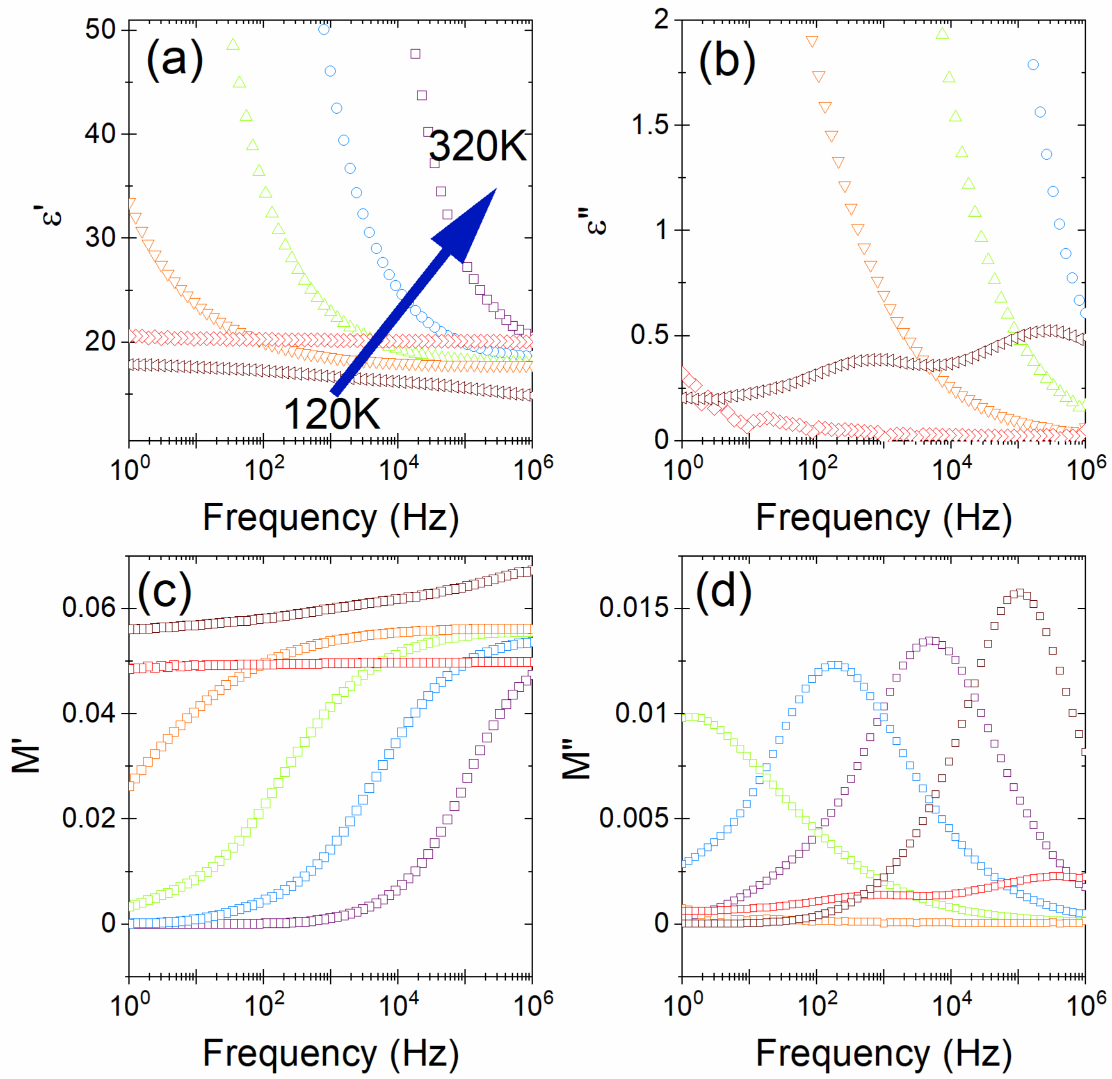

Figure S10. The frequency dependence of dielectric permittivity (a), dielectric loss (b), real (c) and imaginary (d) part of electric modulus spectra of IM_3PbBr_5.

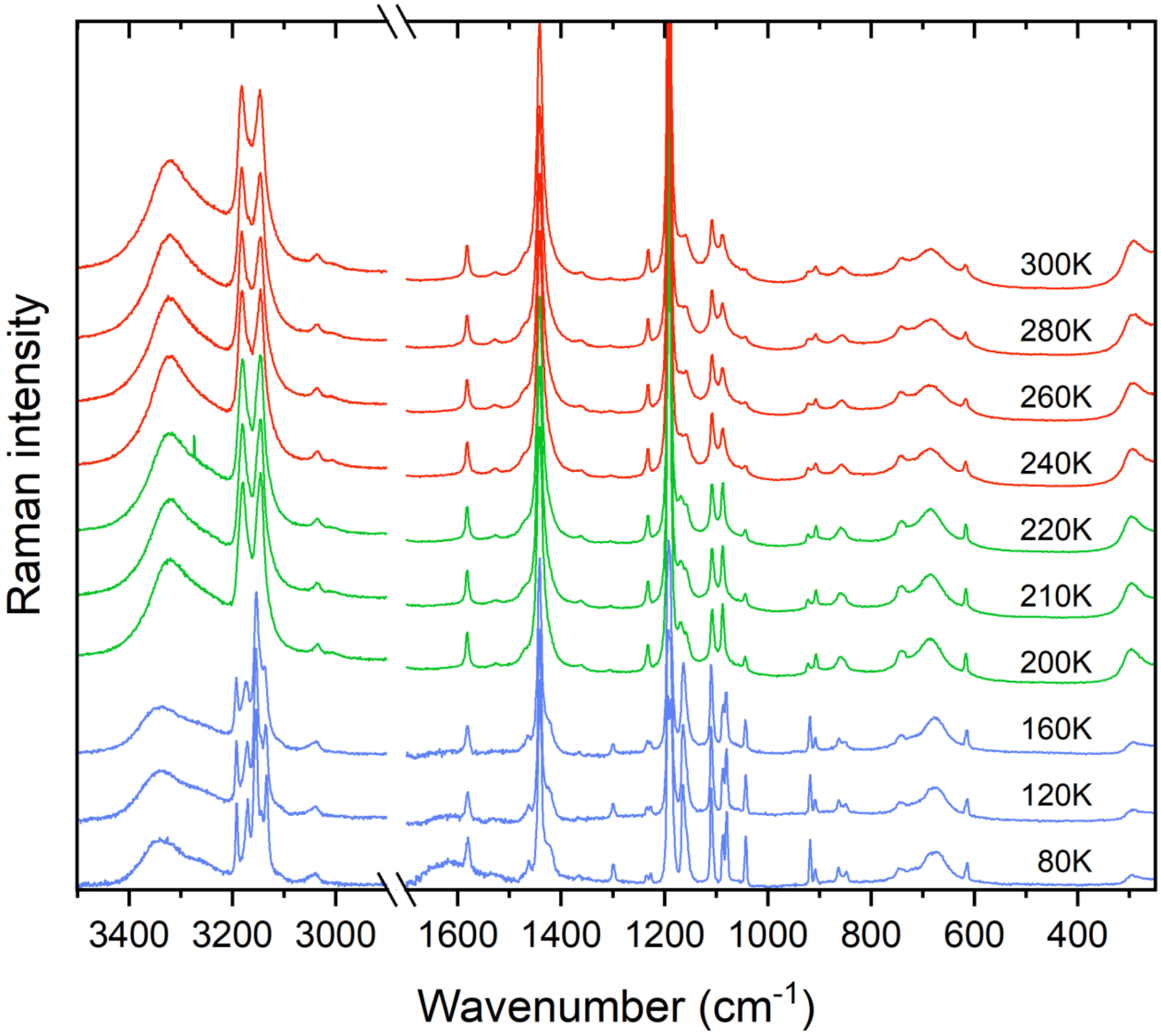

Figure S11. Raman spectra of $IMPbBr_3$.

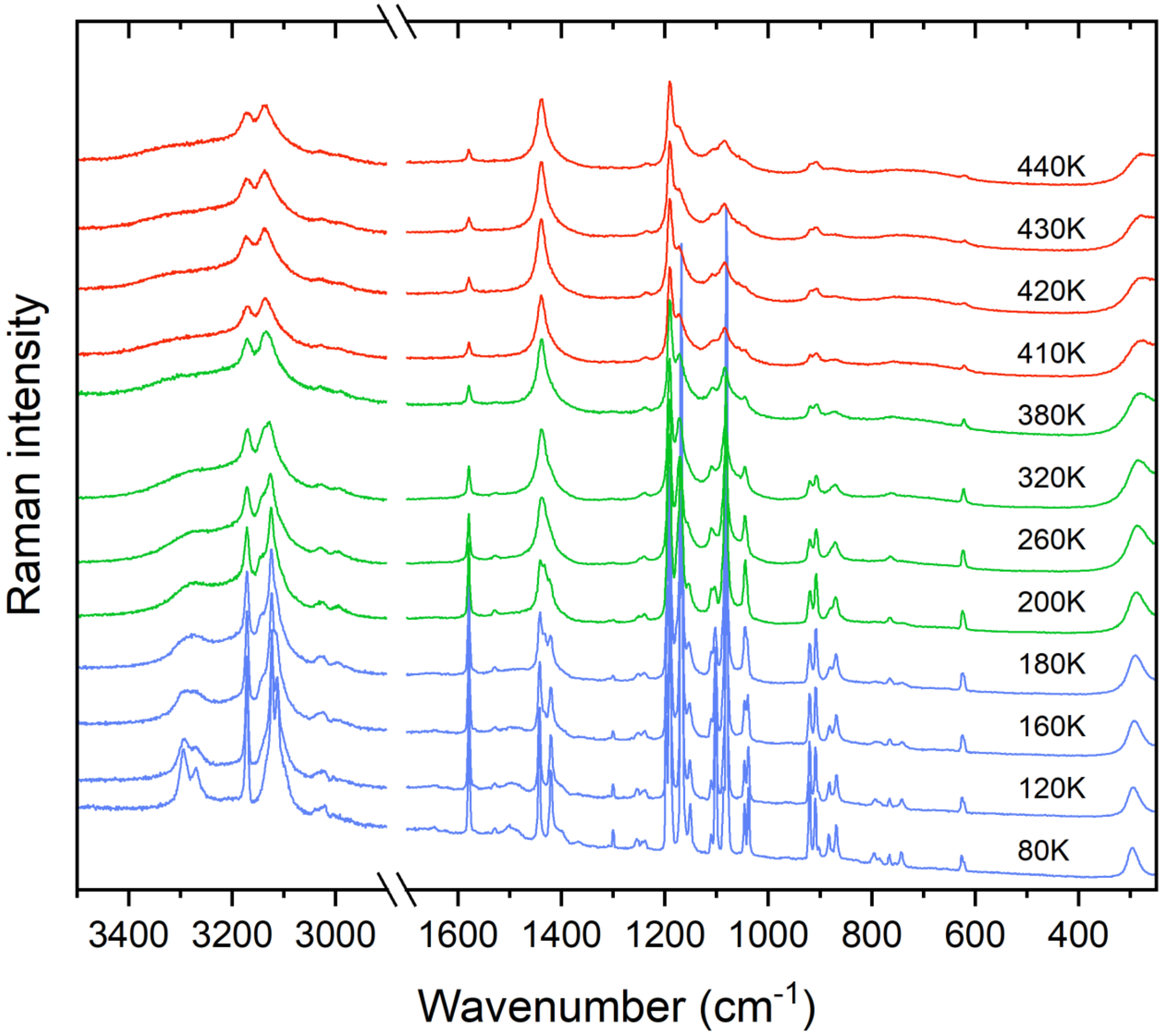

Figure S12. Raman spectra of IM_2PbBr_4.

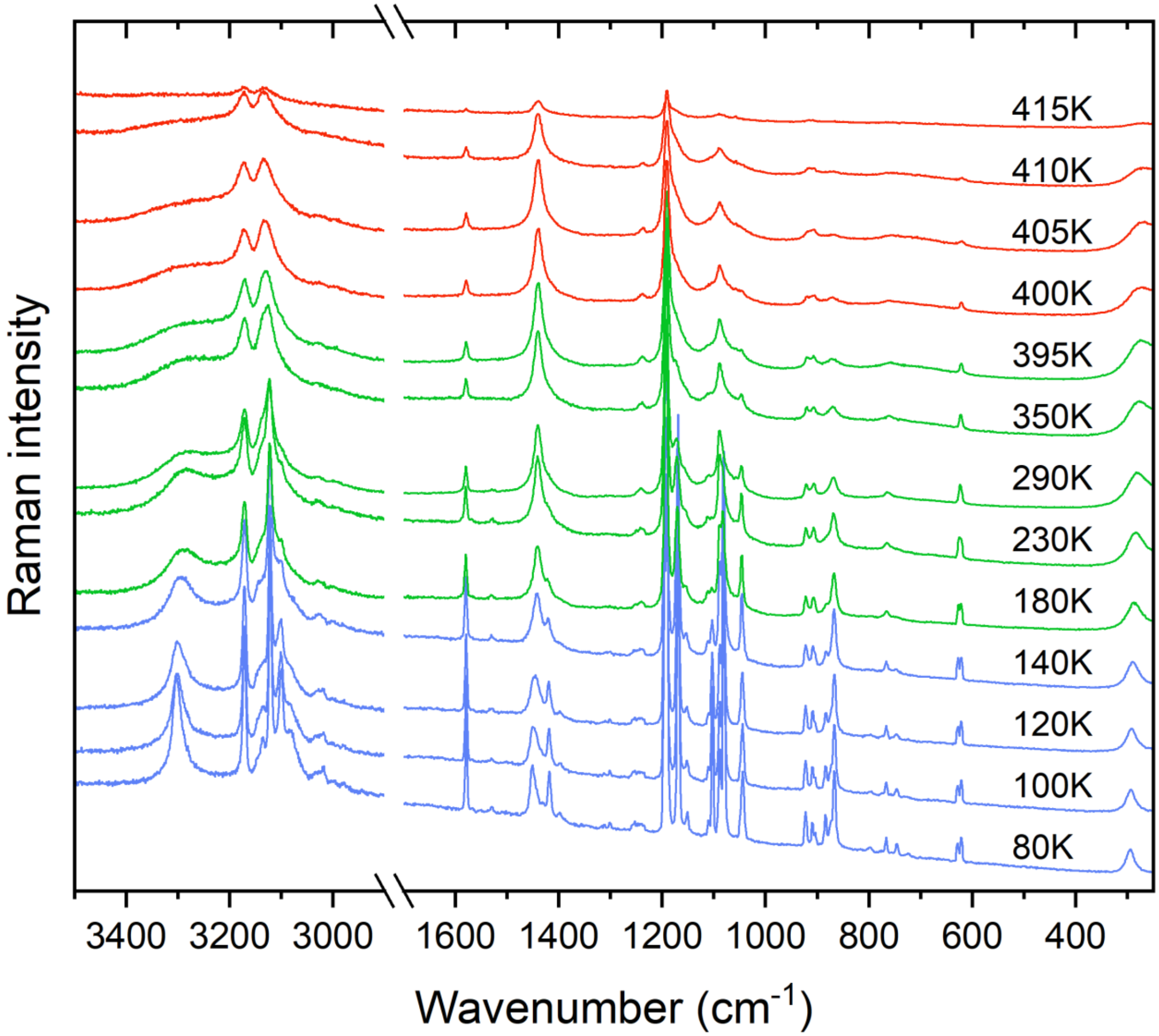

Figure S13. Raman spectra of IM_3PbBr_5.

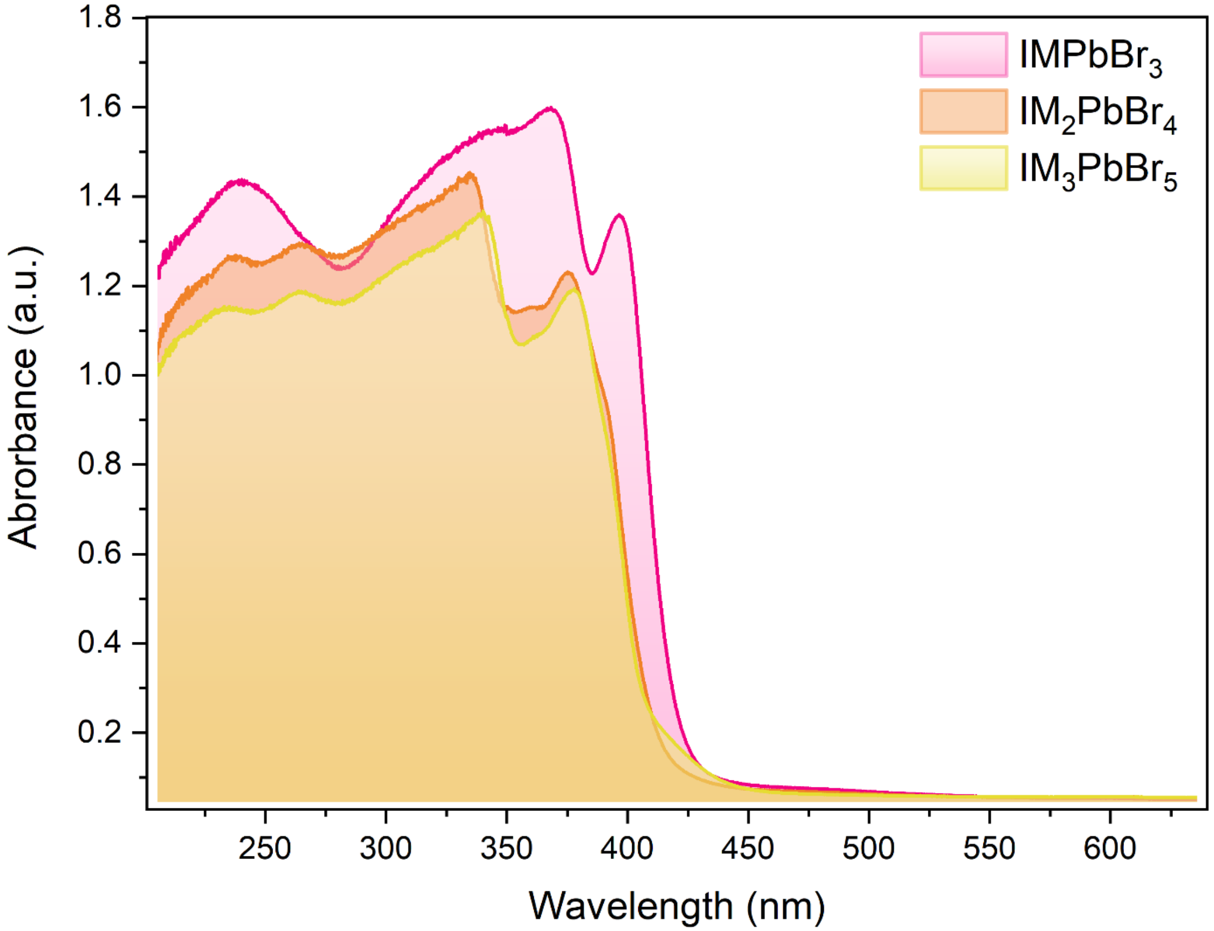

Figure S14. Diffuse reflectance spectra of $IMPbBr_3$, IM_2PbBr_4 and IM_3PbBr_5 recorded at room temperature.

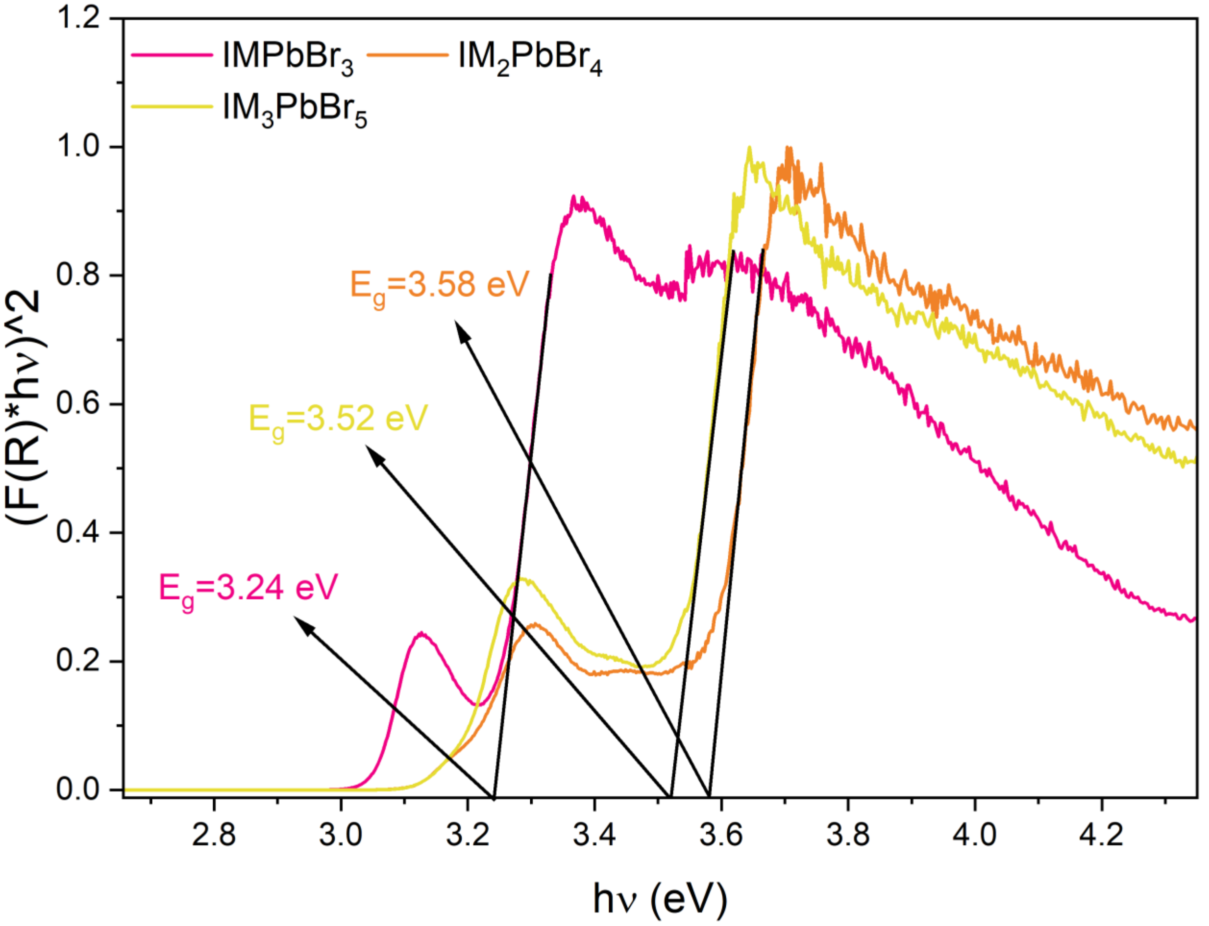

Figure S15. Energy band gaps of the investigated compounds determined using Kubelka-Munk notation.

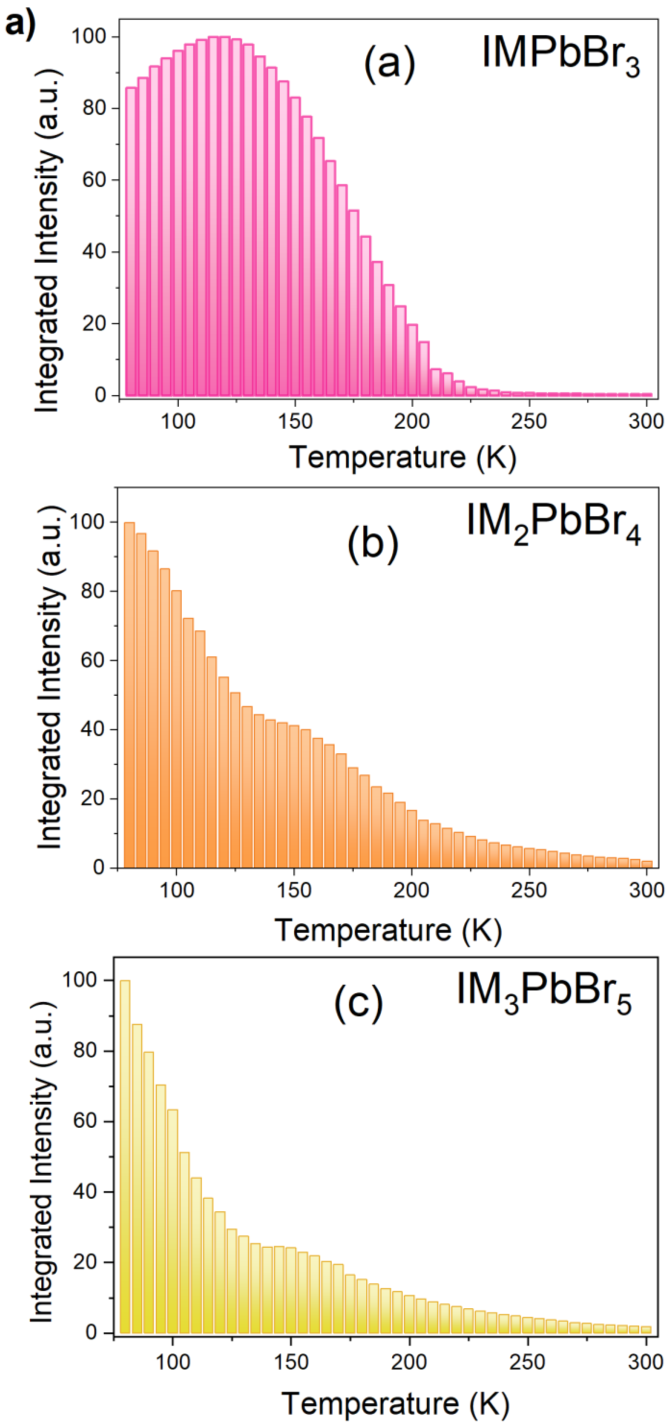

Figure S16. Changes of integrated intensity of PL for (a) $IMPbBr_3$, (b) IM_2PbBr_4 and (c) IM_3PbBr_5 with increasing temperature.

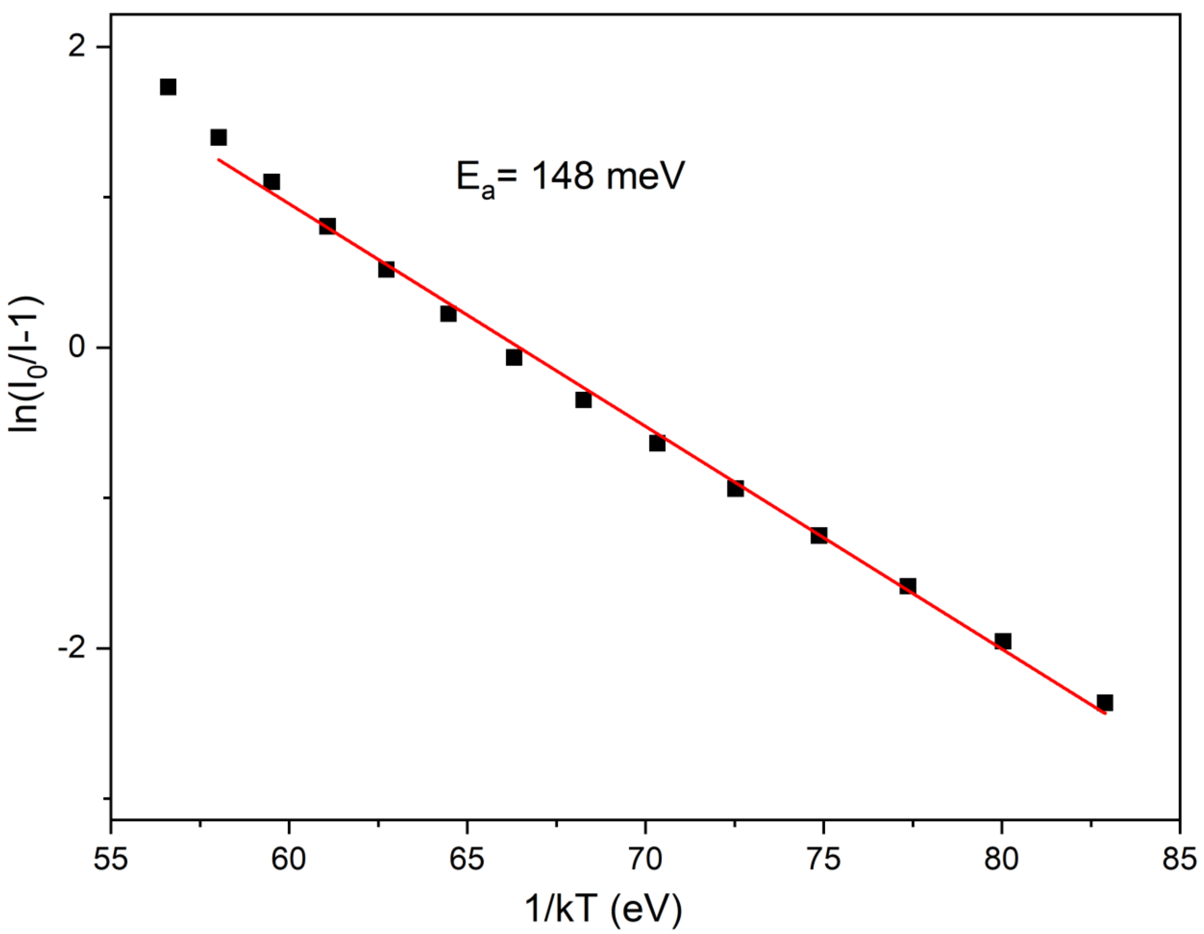

Figure S17. Energy activation for thermal quenching of $IMPbBr_3$.

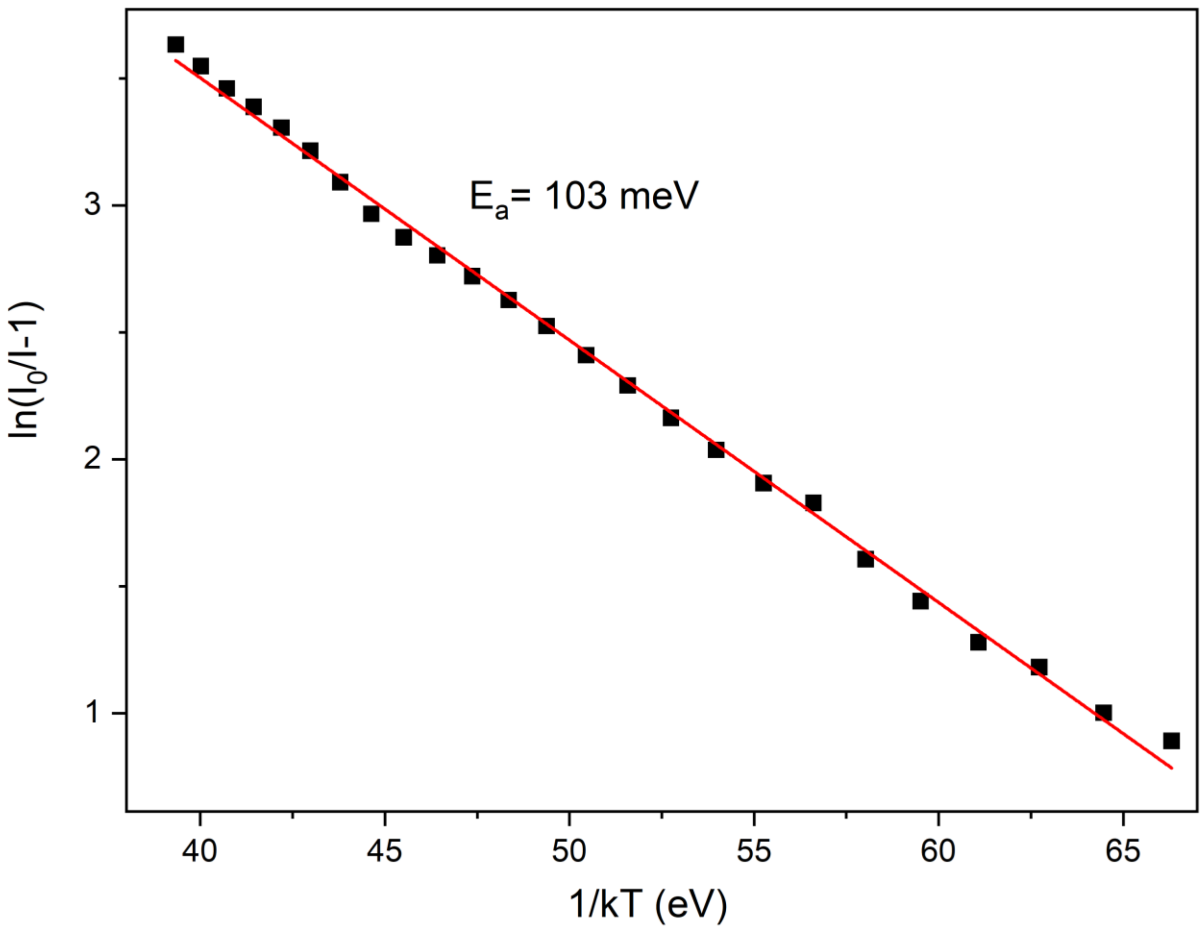

Figure S18. Energy activation for thermal quenching of IM_2PbBr_4.

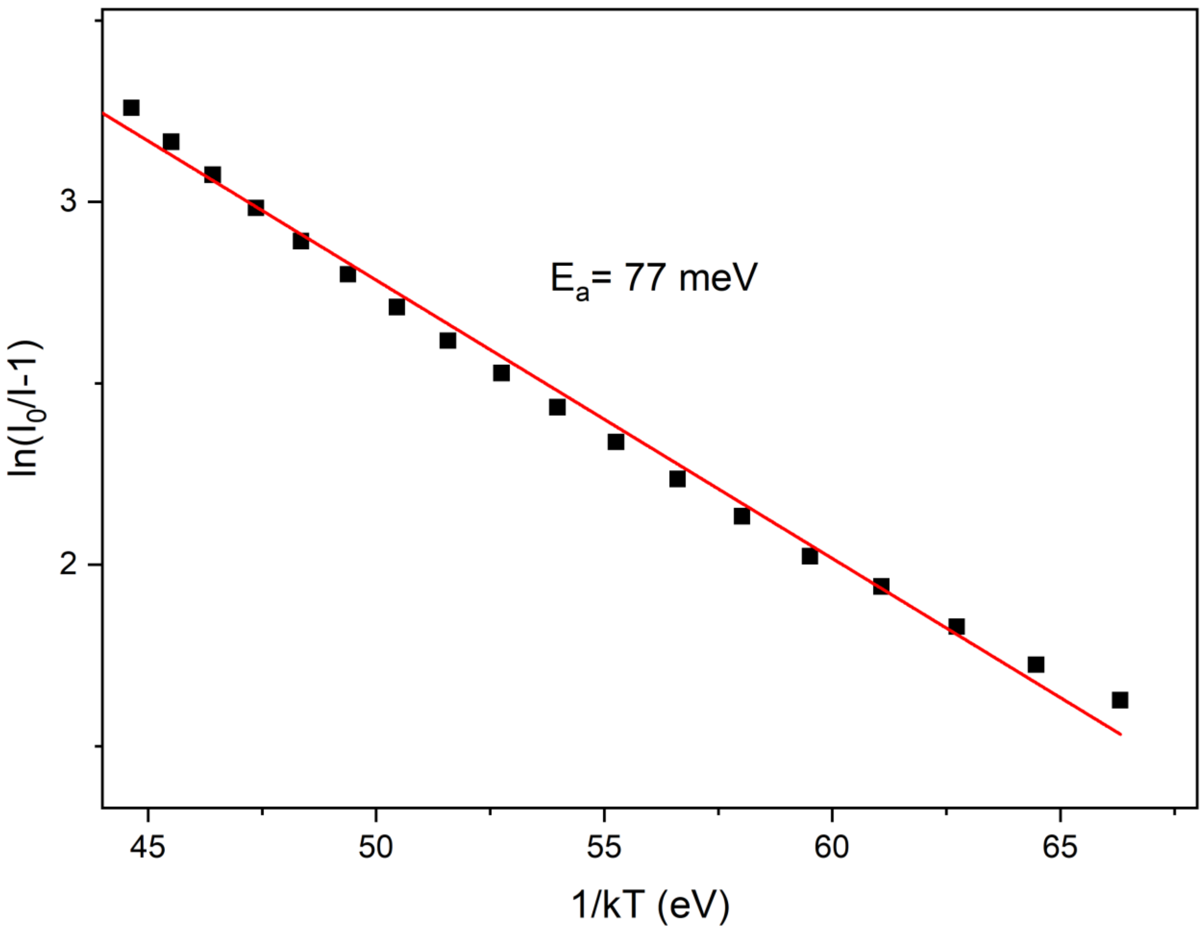

Figure S19. Energy activation for thermal quenching of IM_3PbBr_5.
